# Innovative Formulation Strategies for Biosimilars: Trends Focused on Buffer-Free Systems, Safety, Regulatory Alignment, and Intellectual Property Challenges

**DOI:** 10.3390/ph18060908

**Published:** 2025-06-17

**Authors:** Tomas Gabriel Bas

**Affiliations:** Escuela de Ciencias Empresariales, Universidad Catolica del Norte, Coquimbo 1780000, Chile; tomas.bas@ucn.cl

**Keywords:** biosimilars, high-concentration formulations, buffer-free systems, immunogenicity mitigation, Quality by Design, excipients, Process Analytical Technology, FDA/EMA, intellectual property strategies

## Abstract

The formulation of biosimilar products critically determines their stability, safety, immunogenicity, and market accessibility. This article presents a novel integrative framework for biosimilar formulation that balances scientific, regulatory, and intellectual property dimensions, offering a holistic perspective rarely unified in the literature. It highlights the growing trend toward buffer-free, high-concentration systems that leverage protein self-buffering to improve patient comfort and formulation stability. The article also addresses regulatory flexibility from the FDA and EMA, which allows scientifically justified deviations from reference formulations to ensure pharmaceutical equivalence and minimize immunogenicity. A novelty of this article is its comprehensive analysis of how digital innovations, such as Quality-by-Design, Process-Analytical-Technology, and AI-based in silico simulations, are transforming formulation design and bioprocess optimization to reduce immunogenic risks and enhance bioequivalence. Two important key takeaways emerge: (1) strategic innovation in formulation, especially using buffer-free and high concentration systems, improve product stability and patient tolerability while complying with regulatory standards; and (2) intellectual property challenges, including patent thickets, strongly influence formulation decisions, making early legal-strategic alignment essential for market entry. The article confirms that practical recommendations for the selection of recombinant therapeutic protein formulations can effectively guide developers and regulators toward safer, more efficient, and commercially viable biosimilar products.

## 1. Introduction

Biosimilar medicines are highly sophisticated therapeutic products, specifically developed to replicate biological drugs of origin, while preserving quality, safety, and efficacy, but their intellectual property (patents) has expired [1]. Although they have similar characteristics to the source molecules, these are not identical; however, they are developed with the highest quality standards to match the safety and efficacy of the reference medicine used [2]. The process of developing biosimilars is exceptionally complex because it must address the inherent variability of recombinant proteins and ensure that any differences from the original product are clinically insignificant [3,4]. A fundamental element in the development and production of biosimilars involves the integration of advanced process design, control, and analytical characterization techniques (Quality-by-Design (QbD), Process-Analytical-Technology (PAT), in silico modeling) to anticipate immunogenic risks and optimize bioequivalence, which allows the reduction in uncertainty and simplifies regulatory reviews [5]. In the formulation of biosimilars, there are key components, including stabilizers and excipients, which must also meet the highest quality standards [6,7]. These stabilizers and excipients can profoundly influence the biopharmaceutical profile of the resulting biosimilar, which must also include the shelf life of the new medicine, as well as the method of administration, the efficacy in the treatment of patients, and the potential for immunogenicity [7,8]. Likewise, some innovations such as buffer-free formulations and nanomedicine approaches illustrate how the field of biosimilars is evolving to overcome conventional challenges while improving patient outcomes [9]. All these components, together with the inherent variability of recombinant proteins, play a fundamental role in maintaining the stability and activities of therapeutic proteins, which must be estimated from the beginning of the formulation as fundamental components of the success or failure of the final biosimilar product [10,11,12].

In this complex dynamic, there is one crucial element to consider, that is, the expiration of intellectual property protection, which includes patents. These protect the inventors of the original biological molecules from copying for a period of two decades [1,2]. During this time, the original biological drugs enjoyed a kind of monopoly, which resulted in very high prices for low-income patients and limited their access to effective treatments [13]. Once the patent has expired, it is possible to develop and market biosimilars. Their main attraction lies in their potential to significantly reduce treatment costs without compromising quality standards, as the pharmaceutical companies that manufacture the biosimilars do not have to face royalty payments associated with patent protection or extensive clinical trials with the same duration as the originals [14,15,16,17,18,19,20]. These economic implications related to biosimilars further reinforce their relevance in the market, where they correlate with lower drug prices and increased access to proven therapies that otherwise might have been prohibitively expensive for low-income patients [21,22].

The clinical transition from an original biological drug to a biosimilar can be positively influenced by socioeconomic factors [1]. In fact, since biosimilar products have characteristics similar to their reference counterparts, they are marketed at lower cost, among other things, because of the absence of royalties on intellectual property rights. Likewise, those who access these products generally come from communities with more restricted economic resources (as is the case in many Latin American and African countries); this would facilitate access to first-class medical treatments for these more disadvantaged communities at a much lower cost than IP-protected originals. Furthermore, cost-effectiveness analyses have shown that the strategic implementation of biosimilars could alleviate financial stress on healthcare systems, particularly in economically diverse regions [23,24]. Data reveals that biosimilars can offer substantial reductions in drug spending while maintaining therapeutic efficacy, ultimately improving patient outcomes in chronic disease management protocols [25,26]. Countries that have implemented biosimilars in their treatment protocols have achieved significant savings in final costs, which can be used to improve patient services or to cover more patients within existing budgets [27,28]. Data reveals that biosimilars can offer substantial reductions in drug spending while maintaining therapeutic efficacy, ultimately improving patient outcomes in chronic disease management protocols [25,26]. Countries that have implemented biosimilars in treatment protocols have seen significant final cost savings, which can be redirected to improve patient services or cover more patients within existing budgets [27,28]. This process contributes to the sustainability of healthcare systems by ensuring the quality, safety, and efficacy of biosimilars, optimizing the use of resources by offering more affordable alternatives to low-income patients [29]. However, despite the promising health and economic implications for patients, the complexities of biosimilar manufacturing pose specific challenges. Each biosimilar manufacturer must independently develop specialized cell lines and optimized production protocols capable of consistently producing biologically comparable products [1]. Therefore, the development of biosimilars represents both a scientific achievement and a complex regulatory challenge, highlighting the importance of maintaining strict standards of quality and efficacy as these drugs become increasingly integrated into global healthcare practices [20].

Biosimilars differ primarily from generic drugs in both their production and regulatory approval, as can be seen in the explanation in Table 1.

Therefore, generic drugs are small-molecule compounds synthesized through well-defined chemical processes that allow identical replication of the active pharmaceutical ingredient (API) [3,30,31]. This means that their regulatory approval only requires a demonstration of bioequivalence, that is, that the generic releases the same amount of API into the bloodstream as the reference product, without the need for clinical trials to evaluate efficacy or immunogenicity [1,2].

In contrast, biosimilars are large and structurally complex proteins (typically 10,000 to 300,000 Da) produced in living cell systems using recombinant DNA technology [1]. These biological systems introduce natural variability (e.g., glycosylation, folding, impurities), making exact replication of the originator biologically impossible. Therefore, biosimilars must demonstrate high similarity, and not be identical, to their reference products. Approval is based on a stepwise comparability exercise, beginning with extensive physicochemical and functional characterization of critical quality attributes (CQA), followed by non-clinical and comparative clinical studies, particularly for immunogenicity, pharmacokinetics (PK), and pharmacodynamics (PD).

To ensure therapeutic equivalence, in the United States and Europe, biosimilars undergo a stepwise comparability assessment mandated by the European Medicines Agency (EMA) and the US Food and Drug Administration (FDA), which includes in-depth characterization (e.g., glycosylation, folding), mechanism-of-action confirmation, and clinical immunogenicity studies. Regulatory frameworks from the EMA and FDA apply the ‘totality of evidence’ principle, requiring integrated data from analytical, functional, and clinical domains [2]. Furthermore, biosimilars are not automatically considered interchangeable, since separate regulatory determination is often required for substitution at the drug level, especially in jurisdictions such as the United States. This high regulatory bar reflects the complexity of biologics and the need to ensure therapeutic equivalence despite minor molecular differences. Although biosimilars share identical primary protein sequences and closely conform to three-dimensional configurations critical for biological activity, slight variations in their complex structure inevitably occur due to differences in production processes [32]. These structural nuances require strict regulatory oversight to ensure the therapeutic equivalence of biosimilars with their reference counterparts in terms of efficacy, safety, and quality [33]. Therefore, regulatory frameworks must intervene in rigorous analytical characterization, examining molecular weight, isoforms, impurity profiles, and biochemical attributes using advanced analytical technologies [34,35]. Equally important are comparative clinical studies that evaluate pharmacokinetics, pharmacodynamics, immunogenicity, and overall safety, confirming the clinical comparability of biosimilars with the original biological products [36].

Advanced biosimilar development integrates cutting-edge design, manufacturing, and testing to meet stringent US and EU regulatory requirements. Biosimilars are approved through abbreviated regulatory pathways established by the EMA since 2005 and the FDA through the Biologics Pricing Competition and Innovation Act (BPCIA) of 2009 [1]. These frameworks require robust comparative evidence demonstrating similarity to a reference biologic in terms of quality, efficacy, and safety, while allowing reduced clinical trial burden. By 2023, more than 100 biosimilars had been approved in Europe and the US, including high-impact therapeutic areas such as oncology, rheumatology, and endocrinology [2].

Analytical characterization, using approaches such as mass spectrometry, surface plasmon resonance, and chemometrics, is critical to establish structural and functional similarity to the reference product [37,38]. In manufacturing, different process development strategies, such as the design of experiments and PAT, facilitate important mechanistic control and characterization, ensuring product consistency during scale-up [39,40]. Regulatory pathways are increasingly using innovative analytical data and AI-based models to address various challenges, streamline comparability assessments, and reduce overreliance on large clinical trials [2]. Complementary studies demonstrate that the integration of advanced analytics and digital manufacturing improves both quality control and cost effectiveness in the final production of biosimilars [41].

Formulation innovation in biosimilars, such as altering excipients, adopting buffer-free systems, or increasing protein concentration, is allowed under current regulatory guidelines, but only within the boundaries set by the comparability requirements. These requirements ensure that any modifications do not result in clinically meaningful differences from the reference product. Regulatory agencies assess biosimilarity based primarily on analytical and functional comparability, with clinical testing minimized but still required if residual uncertainty remains. As such, any innovative formulation must be scientifically justified and supported by data demonstrating that the biosimilar’s safety, purity, and potency remain unaffected. This creates a dynamic balance where the developer is encouraged to pursue improvements but must align them with strict comparability thresholds to ensure regulatory acceptance.

Real-time monitoring strategies such as online FTIR and Raman spectroscopy, as part of the PAT framework, ensure that critical quality attributes are maintained within acceptable limits, thus supporting the clinical performance of biosimilars [40,42]. At the same time, QbD initiatives and digital automation, supported by AI analytics, enable biosimilar manufacturers to more efficiently monitor their performance through US and EU regulations to rigorously assess comparability with reference products, optimize process development, and improve future scale-up strategies [2].

This article was developed in response to the growing need for a multidisciplinary framework that integrates formulation science, regulatory flexibility, and intellectual property management in biosimilar development. Unlike the existing literature that addresses these domains in isolation, this research provides a unified perspective to guide scientifically innovative, legally sound, and regulatorily compliant biosimilar formulation strategies, especially considering the challenges posed by buffer-free systems, high concentration formats, and digital design tools.

Based on this background, this article aims to offer a critical and integrative review of biosimilar formulation strategies to improve the accessibility, efficacy, and safety of high-cost biologic therapies. It also offers a critical review of current trends in formulation technologies, with an emphasis on buffer-free systems and high-concentration formulations that optimize protein stability and patient experience. Furthermore, the article analyzes the safety classification of excipients, highlighting FDA and EMA regulations, which authorize justified innovations if biosimilarity is preserved. The article explores the main intellectual property barriers associated with formulation patents and some of the “tangleholds” of rights that delay the market entry of biosimilars. Finally, the article addresses the use of PAT, QbD approaches, and AI-based predictive models to anticipate immunogenic risks and optimize scale-up. This integrative analysis helps guide developers and regulators in the formulation of competitive, safe, and sustainable biosimilars.

## 2. Methodology

For the documentary search, a variant of the PSALSAR methodology was used (Protocol–Search–Appraisal–Synthesis–Analysis–Report) [43]. This methodology offers a rigorous, transparent, and reproducible framework that allows an in-depth evaluation of the most representative systematic review of the literature, allowing the collection of a complete collection of documents related to the aspects investigated and that guide future scientific research [44,45,46,47,48].

The PSALSAR methodology is applicable in the multispectral domain of biosimilar formulation research, where technological, regulatory, and legal frameworks converge. Its structured approach addresses the complex interactions between pharmaceutical formulation, regulatory science, and intellectual property, facilitating a comprehensive review of the literature that enhances methodological rigor and narrative cohesion [3,5]. Unlike traditional systematic reviews focusing solely on clinical outcomes, PSALSAR supports an argument-driven synthesis that effectively integrates formulation-related aspects such as buffer selection, along with regulatory considerations such as QbD and PAT [3]. The distinction of this methodology lies in its adaptability, which allows it to synthesize various types of content (scientific, regulatory, and legal) while allowing deeper interpretative analysis that goes beyond mere data aggregation, ultimately allowing for in-depth research into biosimilar formulations.

### Keywords and Document Identification Process

The methodological process began with an exploratory phase based on the research objective, in which representative keywords of the thematic domain addressed in this systematic review were defined [49]. These keywords were selected for their high scientific relevance, their ability to effectively delimit the universe of study, and their methodological usefulness in establishing inclusion and exclusion criteria, in accordance with the principles of transparency and reproducibility established by the PRISMA methodology. To guarantee effective and specific document retrieval during the identification phase, a set of strategic keywords was defined that allowed the search to be precisely and systematically guided through scientific databases and search engines with high scientific visibility, such as Scopus, Web of Science, Science Direct and Google Scholar, Core Collections, Science Direct, Compendex, Derwent, Google Scholar, Innovation Index and GeoIndex. These sources were complemented by interdisciplinary research tools that expanded the coverage and precision of the results obtained. To this end, both controlled and uncontrolled terms were integrated using Boolean operators and truncation strategies. Keywords used were the following: “Biosimilars”; “Biology”; “Biotechnology”; “High-concentration formulations”; “Buffer-free systems”; “Immunogenicity mitigation”; “ Excipients”; “Quality by Design (QbD)”; “ In silico simulations”; “Process Analytical Technology (PAT)”; “Food and Drug Administration (FDA)”; “ European Medicines Agency (EMA)”; “Intellectual property strategies (IP)”; “Artificial Intelligence (AI)”. Each of these words was selected for its relevance using “AND” and “OR” to capture the central dynamics of the object of study, covering regulatory, technological, clinical, and legal aspects linked to the development and formulation of biosimilars. Likewise, they were formulated in English to maximize coverage in the high-visibility scientific databases used.

Table 2 provides a more detailed analysis of the inclusion and exclusion criteria used to select the references ultimately used in the research. These criteria are established prior to the bibliographic search based on the identified keywords to reduce bias and ensure that the document selection process is systematic and reproducible.

**Figure 1:**
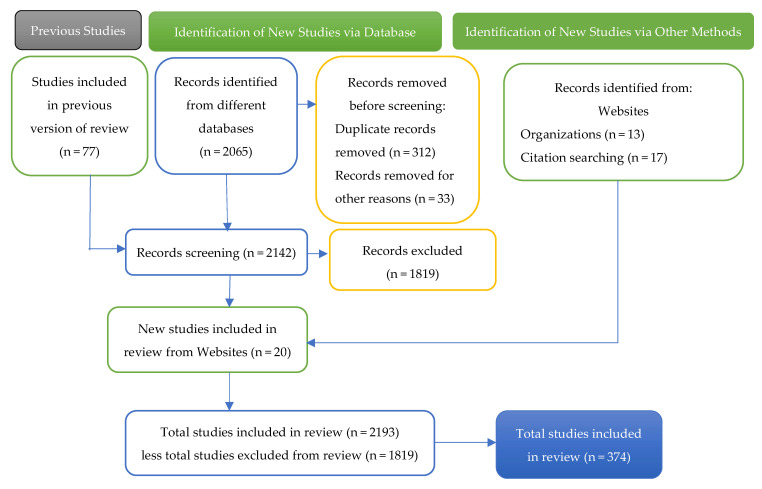
PRISMA-based search process.

The review process incorporates a flow chart represented in Figure 1, which illustrates the selection procedure, including the number of studies identified, selected, and involved, as well as the reasons for exclusion at each stage. The documentary strategy included a systematic review from 2019 to 2025, which resulted in a repository of 2193 documents (32 websites). This repository offers a representative sample of the state of the art on the most innovative research topic and integrates perspectives from different disciplines with an emphasis on the formulation of biosimilars in the current and future biopharmaceutical context.

## 3. Design, Manufacturing, and Analytical Characterization of Biosimilars

The design, manufacturing, and subsequent analytical characterization of biosimilars play an important role in establishing confidence in these products and in ensuring that critical quality attributes reflect those of the reference product like observed on Table A1. Biosimilar development involves not only replicating the primary amino acid sequence but also mimicking post-translational modifications such as glycosylation, which are essential to maintain clinical efficacy and safety and thus ensure that CQAs remain within acceptable ranges [50,51]. Continuous improvements in process design, including real-time monitoring and QbD initiatives, have become a cornerstone of biosimilar development [52]. The application of QbD in conjunction with the design of experiments (DoE) establishes defined control strategies and design spaces, ensuring consistent process performance and product reproducibility [37,53,54]. The use of PAT and real-time monitoring, complemented by advanced chemometric analysis, facilitates an accurate assessment of both upstream and downstream processes [55,56]. As manufacturing processes become increasingly automated and deep data-driven, the integration of machine learning for pattern recognition further improves the control of the entire process [57].

Controlled downstream processing is essential for biosimilar manufacturing, as it ensures that chromatographic purification meets strict quality requirements [58]. Advanced analytical technologies, such as ultrafiltration/diafiltration and multimodal chromatography, facilitate the high-resolution separation of aggregates and impurities from the process, thus improving the purification accuracy during scale-up [39,59].

### 3.1. Analytical Characterization and Processing Strategies of Biosimilars

Post-translational modifications (PTMs), such as glycosylation, phosphorylation, deamidation, and oxidation, significantly influence the stability, efficacy, and immunogenicity of biologics, including biosimilars. Unlike conventional drugs, biosimilars are produced in living cells, leading to variability in PTM profiles, which can impact critical aspects such as receptor binding, clearance rates, and antibody-dependent cellular cytotoxicity (ADCC) [60].

The achievement of consistent PTM profiles is complex due to the different expression systems and the variable bioprocessing conditions, which require advanced analytical techniques such as liquid chromatography tandem mass spectrometry (LC-MS/MS) and capillary electrophoresis for thorough characterization [61]. Immunogenicity remains a concern, as even slight differences in PTM can provoke adverse immune responses, potentially compromising therapeutic effectiveness [62]. Even small differences in glycosylation profiles (e.g., sialylation or fucosylation) can alter pharmacokinetics and affect receptor binding affinity, clearance rates, and effector functions such as antibody-dependent cell cytotoxicity (ADCC) [63].

Achieving consistent PTM profiles is a major challenge due to the following:

- Differences in expression systems (e.g., CHO vs. HEK293);
- Cell culture conditions, including pH, oxygen, and nutrients;
- Bioprocessing variables, such as purification methods or buffer composition.

These factors mean that biosimilar developers must implement advanced analytical technologies (e.g., LC-MS/MS, capillary electrophoresis) to rigorously characterize PTMs and demonstrate high similarity to the reference product [64,65].

Immunogenicity is a major concern precisely because even minor differences in PTMs or impurities can elicit an unwanted immune response. These responses may range from mild infusion reactions to neutralizing antibodies that compromise therapeutic efficacy, or, in severe cases, cross-react with endogenous proteins, as historically occurred with epoetin alfa.

Regulatory bodies (FDA and EMA) demand comprehensive comparative studies that assess immunogenicity, recognizing the importance of minimizing aggregates and choosing appropriate excipients [66,67,68].

The formulation and long-term performance of biosimilars are crucial to their therapeutic success in a highly competitive industry. Molecular design in biosimilar development emphasizes engineering expression systems that closely resemble the reference biologic. Techniques such as optimizing codon usage and designing suitable signal peptides are vital to achieve biochemical equivalence with the innovator [69]. However, due to the inherent complexity of biologics, the achievement of identical molecular structures is implausible. Instead, a rational engineering approach focuses on replicating functional domains that dictate therapeutic efficacy [70].

Reproducing PTMs, especially glycosylation, is particularly challenging. As PTMs significantly influence the pharmacokinetics and immunogenicity of biologics, meticulous control over production conditions is necessary [71]. Advanced analytical techniques, including LC-MS and HILIC, facilitate comparative glycosylation profiling, which is essential to establish biosimilarity [72]. Furthermore, strategies such as the use of nanoparticles for stabilization hold promise to enhance the delivery and efficacy of sensitive biosimilar products [73].

Molecular design in biosimilar development requires meticulous attention to protein structure and function, particularly focusing on how recombinant expression systems and upstream processes can replicate the characteristics of reference biologics [74]. This intricate process emphasizes the role of signal peptides in facilitating proper protein folding, the optimization of codon usage for effective expression in host cells, and the selection of appropriate expression systems to closely align with the glycosylation profiles of the reference products. While achieving complete molecular identity is unattainable due to the inherent complexities of biologic synthesis, molecular engineering strategies aim to reproduce the key functional domains critical for therapeutic activity and receptor interaction [2,75].

The creation of biosimilars must account for variations in PTMs, with glycosylation being one of the most significant challenges faced in this developmental landscape. Glycans, which are carbohydrate structures attached to proteins, can affect the biological function, stability, immunogenicity, and half-life of the resultant biosimilars. This complexity arises from the sensitivity of glycosylation to cellular environments, including cell type, medium composition, culture conditions, and purification processes, which can dramatically influence glycan structure [76]. As such, developers utilize orthogonal methods alongside extensive CQA modeling to verify that the PTM profiles align closely with those of the reference biologic [77,78].

Advanced analytical techniques such as liquid chromatography mass spectrometry (LC-MS), nuclear magnetic resonance (NMR), and high-resolution chromatography play critical roles in ensuring that the molecular architecture and post-translational landscapes of biosimilars are consistent and comparable to their reference products. These methodologies facilitate detailed examination and characterization of glycosylation patterns and other critical quality attributes, supporting rigorous testing and validation protocols that are essential for regulatory approval [72,79]. For example, methods such as mass spectrometry allow precision in identifying glycosylation sites and structures, thus confirming conformity to established specifications set by authorities such as the FDA [68].

The variability inherent in PTMs necessitates a robust approach to verifying these modifications, as even minor discrepancies can translate into considerable differences in therapeutic efficacy and safety profiles. Researchers have proposed using sophisticated algorithms and automation to streamline the analyses of glycosylation and other PTMs during the biosimilar development process. This could improve the reliability of assessing biosimilarity and contribute to more consistent product quality [2,80].

Although challenges remain with respect to glycosylation consistency, advancements in nanomedicine also present exciting avenues for addressing the stability and delivery of biosimilars. Nanotechnology, which includes approaches such as nanoparticle encapsulation or liposomal carriers, serves to protect sensitive biologics from environmental degradation while enabling sustained release mechanisms. Such strategies can potentially mitigate issues related to immunogenicity while also improving pharmacokinetic profiles [81]. The ongoing integration of nanotechnology into biosimilar formulations reflects a growing recognition of the potential it holds for the next generation of therapeutic interventions.

Moreover, through ongoing research into glycomic profiling and enhanced production techniques, it is increasingly plausible to generate biosimilars that mimic their originators more closely. This includes the manipulation of culture conditions, bioreactor designs, and even the use of plant-based production systems that facilitate the expression of complex glycosylated proteins [69,82]. Innovative analytical strategies will continue to evolve, providing pathways to not only replicate but also optimize these characteristics dynamically throughout the development process [83].

The regulatory landscape remains a key component in the development and approval of biosimilars. Authorities are focused on ensuring that these products not only meet safety and efficacy benchmarks but also provide transparency and consistency in quality control [84]. With continuing advancements in biotechnology and analytical methods, along with strong regulatory frameworks, the outlook for biosimilars appears promising. The convergence of rigorous analytical methodologies and innovative molecular design will be crucial in the delivery of clinically effective and safe biosimilar therapies to patients around the world [85,86].

The analytical characterization of biosimilars has become increasingly sophisticated with the implementation of orthogonal techniques that investigate various aspects of molecular structure [37]. This employs an integrated ‘totality of evidence’ strategy that uses high resolution mass spectrometry, as well as multidimensional LC-MS (or liquid chromatography coupled to multidimensional mass spectrometry), which is an advanced analytical technique that combines several chromatographic separation steps with mass spectrometry (MS) to achieve a more precise, in-depth, and complete characterization of complex chemical mixtures, such as proteins, metabolites, lipids or natural extracts [87]. LC-MS methods enable high-resolution analysis of intact proteins, peptide mapping, and glycan profiling in a single workflow [88]. This multiplexing capability is essential for biosimilars, where even small differences in post-translational modification patterns can have significant immunogenic implications. Orthogonal approaches ensure that differences in charge variants and glycan profiles, which are critical quality attributes, remain within acceptable ranges as required by regulatory authorities in both the United States and Europe [37,50,89]. Advanced nuclear magnetic resonance spectroscopy (NMR) techniques, bidirectional heteronuclear (bidirectional heteronuclear NMR or bidirectional heteronuclear correlation NMR), which is a specialized NMR technique that correlates nuclei of different chemical elements to more accurately analyze molecular structure, allowing to analyze how distinct nuclei connect in both directions within a molecule [21]. All these techniques facilitate a deeper understanding of the chemical structure and are especially valuable in complex structural analyses such as those of biomolecules, improving spectral interpretation and assignment to reveal subtle variations in glycosylation and higher order structures [90,91,92]. Furthermore, statistical methods such as bootstrapping tests have improved confidence in biosimilarity assessments, ensuring batch-to-batch consistency and mitigating process-induced variations [93]. Together, these advanced physicochemical and functional analyses support the reliable development of biosimilars by providing a robust framework for confirming safety and efficacy at the molecular level [94].

### 3.2. Different Strategies and Processing Advances in the Manufacture of Biosimilars

Advances in manufacturing also include the adoption of continuous processing strategies and real-time release testing enabled by PAT. Advances in manufacturing have benefited significantly from the integration of miniaturized PAT into continuous processing systems [95]. These advances enable the real-time detection of critical quality attributes such as protein aggregates and glycosylation variants, facilitating the implementation of immediate corrective actions and reducing batch-to-batch variability [40,96]. Incorporation of online tools, such as Raman spectroscopy, facilitates process monitoring, ensuring that design deviations are addressed quickly [97,98]. Biosimilar analysis similarity assessments are now recognized as a comprehensive exercise that focuses not only on physicochemical properties but also on functional performance of the molecule [3]. Some novel methods were used, including multifaceted bioassays and cell-based studies, to assess biological activity against the reference product. Such studies are vital for the acceptance of biosimilars by regulatory authorities in both the US and European markets as they establish a direct link between manufacturing quality and clinical outcomes. This ‘totality of evidence’ approach ensures that any subtle manufacturing differences do not translate into clinically meaningful differences.

QbD is integral to biosimilar manufacturing, as it emphasizes the identification of critical quality attributes (CQAs) and their alignment with critical process parameters (CPPs). This strategic change enhances the robustness of the production process, mitigating variability and ensuring that the biosimilar meets regulatory requirements [99,100]. Furthermore, PAT improves QbD by allowing continuous real-time monitoring of critical parameters during manufacturing, ensuring consistent quality between batches [101].

Glycosylation is crucial for the characterization of biosimilars, significantly influencing their pharmacokinetics, efficacy, and immunogenicity. Regulatory authorities require comprehensive glycan profiling through advanced analytical techniques to establish bioactivity and comparability with the reference product, reinforcing the importance of glycosylation in the evaluation of biosimilars [53]. Collectively, QbD, PAT, and glycosylation profiling are pivotal in supporting the development of safe and effective biosimilars that meet stringent regulatory standards.

Furthermore, advanced end-point assays such as multiangle light scattering (MALS), differential scanning calorimetry (DSC), and dynamic light scattering (DLS) have improved the ability to monitor biosimilar thermal and colloidal stability, as well as aggregates and subtle conformational changes induced by manufacturing stress, thus ensuring batch consistency and strengthening quality control during process validation [102].

Orthogonal analytical approaches are vital in verifying biosimilarity due to the complexities inherent in biological products. These approaches utilize multiple independent methods, such as DSC, MALS, and DLS, to assess critical attributes of biosimilars. When employing these diverse techniques, the risk of bias of a single method is mitigated, thus strengthening the validity of claims of biosimilarity [103].

DSC measures the thermal transitions of proteins, providing insight into their thermal stability and folding integrity, which are essential for confirming identical functional characteristics between a biosimilar and its reference product [104]. MALS, combined with size-exclusion chromatography, enables the determination of absolute molecular weight and aggregation state, critical for ensuring physicochemical equivalence [105]. DLS focuses on the size distribution and colloidal stability of particles, allowing for early detection of aggregation phenomena that could affect therapeutic efficacy [37,103].

Together, these techniques provide a comprehensive dataset that supports regulatory compliance and the totality-of-evidence approach mandated by regulatory agencies, ultimately facilitating the biosimilar approval process [5,106].

Integration of these orthogonal assays with high-resolution structural characterization methods, particularly X-ray crystallography and cryoelectron microscopy, further allows detailed mapping of the 3D conformation of the protein, crucial for verifying biosimilarity and shelf-life stability [107,108].

**Figure 2:**
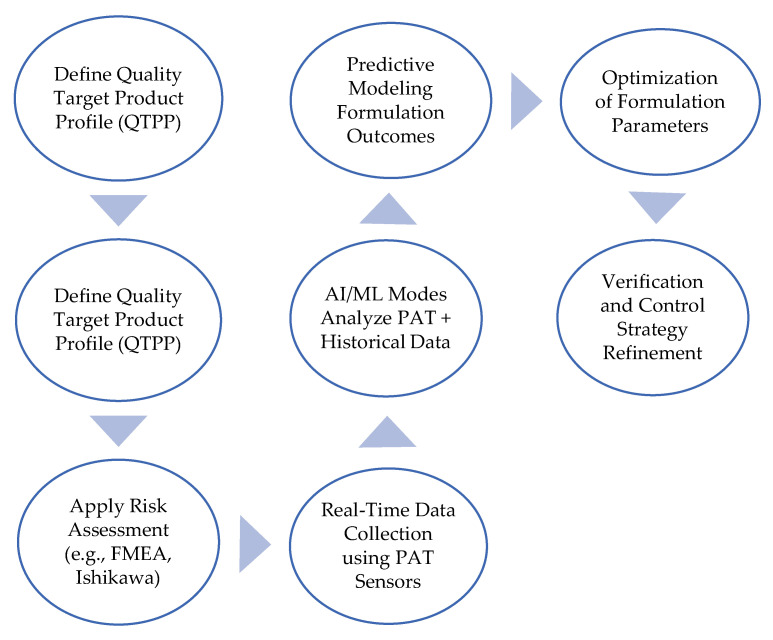
Integration of QbD, PAT, and AI Rational Biosimilar Formulation Design.

To facilitate a practical understanding of how digital tools integrate with regulatory-aligned formulation design, Figure 2 illustrates a stepwise flow of QbD–PAT–AI integration in biosimilar development. This system approach enables real-time feedback, rational formulation optimization, and predictive analytics for critical quality attributes.

Table 3 complements Figure 2, allowing a more detailed understanding of the sequential steps and digital tools involved in the workflow of integrated biosimilar formulation with QbD-PAT-IA.

### 3.3. Integrating AI and Machine Learning into the Biosimilar Development Process

The integration of AI and machine learning is transforming biosimilar development by analyzing large-scale manufacturing and datasets to predict critical quality attributes and detect process anomalies early [2,37,109]. AI-based systems improve process design by facilitating predictive quality assessments like those developed for originator manufacturing, now extending to analytical similarity of biosimilars and optimization of culture media [110]. Moreover, by evaluating parameters such as glycosylation patterns, these computational tools support robust process validation and agility in production environments, ensuring that product quality consistently meets regulatory standards [111,112]. AI facilitates real-time process monitoring and control in biosimilar production by integrating advanced analytics with PAT systems to analyze bioprocess data streams, improving adaptive feedback mechanisms that minimize variability and optimize yields [2,113]. Although still in the early stages, the convergence of these advanced techniques promises to optimize process variables, thereby reducing deviations and accelerating scale-up while advancing quality control measures in biosimilar development [37,114]. Integration of digital tools and AI in the formulation of predictive models and in silico simulations represents a transformative advance that spans multiple sectors, from the discovery of new drugs to the optimization of industrial processes. Recent advances in AI, such as the multihead attention-based drug repurposing recommendation network (MRNDR) model, demonstrate the potential of multihead attention mechanisms in the prediction of complex biological interactions [115]. The model uses a large-scale dataset and advanced machine learning techniques to predict drug-disease relationships, achieving state-of-the-art performance metrics. Although the primary focus is drug repurposing, the methodologies employed, such as multi-head self-attention mechanisms and weighted representation distance scoring, are relevant to biosimilar formulation optimization. These techniques can improve the prediction of protein stability, aggregation propensity, and immunogenicity, which are critical factors in the development of biosimilars. While MRNDR is primarily applied to drug repurposing, its underlying architecture can be adapted to predict critical quality attributes in biosimilar formulations, thus enhancing the efficiency and accuracy of formulation development processes. This convergence is based on the ability to create digital twins that replicate the behavior of real systems in virtual environments, allowing the simulation, analysis, and prediction of different scenarios without incurring high costs and risks inherent to physical tests [115,116,117]. In biosimilar development, a digital twin is used, representing a virtual real-time replica of the bioprocess derived from mechanistic models and sensor data, to simulate and predict the impact of process changes on CQAs before execution, streamlining the QbD approach [101,118].

Deep learning models, particularly convolutional neural networks (CNNs) and recurrent neural networks (RNNs), play a significant role in forecasting protein aggregation during formulation processes. By analyzing large datasets of molecular structures and properties, these models can effectively identify aggregation-prone regions, helping guide excipient selection and thus mitigate immunogenicity risks and improve biosimilar stability over time [2,119]. In the pharmaceutical field, the use of transformer-based models has been developed to estimate drug-target interactions, significantly optimizing the discovery and validation process of potential compounds [120]. Approaches used in silico for the design and prediction of aptamers improve efficiency in the identification of biomarkers and therapeutic optimization [121]. In silico simulations allow exploring multiple scales, from molecular processes to macrolevel systems, using mathematical modeling methods combined with AI algorithms. These techniques have been extended to the simulation of complex interactions in antibody-based therapies, where the integration of multiscale models is crucial to understand the relationship between molecular structure and process behavior at the production level [122]. In silico simulations are increasingly pivotal in the design of aptamers and in the optimization of therapeutic processes. By employing molecular coupling and dynamic simulation algorithms, researchers can accurately predict aptamer-target interactions, refine binding affinities, and enhance target specificity without extensive physical experimentation, thus streamlining the development of these biomolecules for therapeutic purposes [123]. This modeling approach not only reduces costs and time but also helps in the preassessment of aptamer viability as diagnostic tools or therapeutic agents. Predictive modeling is crucial for scaling up biosimilar manufacturing because pilot-scale performance often does not translate directly to larger scales. Mechanistic and statistical models simulate critical bioprocess variables, such as shear stress and oxygen transfer rates, enabling developers to anticipate quality attributes and operational challenges before industrial implementation [124]. This predictive capacity minimizes risks, improving the reliability and efficiency of scaling operations. Single-use bioreactors enhance the safety of biosimilar production by significantly reducing cross-contamination risks associated with traditional systems. Their design eliminates the need for extensive cleaning and sterilization procedures between batches, promoting contamination control and operational efficiency, particularly in multiproduct facilities [125]. By facilitating rapid changeovers and offering greater flexibility, single-use systems support agile manufacturing practices essential for modern biosimilar production. Integrating ‘in silico’ approaches in drug discovery not only reduces the time and cost of experimental testing but also allows the prediction of complex behaviors and iterative adjustment of designs [126]. In this context, the application of silico models not only allows simulation of complex scenarios at different scales but also improves the efficiency and effectiveness of the development process by reducing experimentation time and associated costs and facilitating early validation of hypotheses before physical implementation [127,128].

Advances in deep learning, particularly in the application of graph neural networks (GNNs), have proven to be very important in modeling molecular structures and predicting their properties with high precision [129,130]. Similarly, a multiscale graph neural network model that integrates features at different levels of the molecular structure to predict properties with comparable or superior performance compared to other methods [131]. These approaches allow for a detailed characterization of the molecular structure, offering a solid basis for optimization in the development of biosimilars [128]. The use of GNN-based approaches to map protein-protein interfaces allows us to optimize the compatibility and functionality of biosimilars by predicting critical molecular interactions [132].

Another crucial aspect is the improvement in the accuracy of predictive models through the fusion of deep learning techniques. learning e, 3D structural information. By incorporating graph neural networks together with models based on three-dimensional structure, it is possible to predict the binding affinity between ligands and proteins more accurately [133]. This methodology is especially relevant in the context of biosimilars, where optimization of molecular interactions directly influences therapeutic efficacy and similarity. Similarly, encoder–decoder models improve target-directed design, accelerating the identification of desired molecular profiles and reducing uncertainty at critical stages of development [134].

Integration of digital platforms and AI tools into biosimilar formulation workflows enhances molecular stability and design efficiency. For example, simulations such as GastroPlus^®^ and ADMET Predictor^®^ by Simulations Plus facilitate pharmacokinetic predictions and excipient compatibility assessments, which are crucial for biopharmaceutical developments [135]. AI advancements, particularly AlphaFold’s ability to predict protein structures, are increasingly vital for understanding protein stability under varying conditions [80]. Additionally, platforms such as IBM Watson, DeepChem, and BioPharma Finder™ are utilized for real-time data analysis, optimizing process parameters, and supporting Quality-by-Design (QbD) principles by aligning formulation attributes with CQAs [136].

Furthermore, recent studies have suggested that such digital approaches contribute to accelerating biosimilar development by streamlining regulatory compliance processes, thus improving clinical effectiveness and safety profiles [7]. This technological evolution not only boosts operational efficiency but also mitigates risks in formulation processes, making a strong case for the adoption of integrated digital solutions in pharmaceutical applications [137].

The integration of digital platforms and AI-driven tools into biosimilar formulation workflows significantly improves the design, stability, and risk assessment of biosimilars. Tools such as Schrödinger’s BioLuminate can predict aggregation-prone regions using molecular dynamics simulations, thus increasing predictability in formulation outcomes [138]. Furthermore, Sartorius’ MODDE^®^ software provides a design-based optimization approach based on experiments (DoE), merging empirical data with predictive analytics to effectively refine formulation variables [2]. Simulations Plus offers GastroPlus^®^ and ADMET Predictor^®^ to predict pharmacokinetic behavior and excipient compatibility. In the AI domain, AlphaFold’s capability in predicting protein structures is transformative, facilitating formulation development by anticipating structural stability challenges [138]. The deployment of IBM Watson and machine learning tools such as DeepChem enables advanced data mining and trend analysis, which aligns with the QbD framework that emphasizes rational selection of formulation attributes based on critical quality attributes [2]. Such innovations not only streamline workflows but also contribute to greater efficiency in drug development, offering a pathway to more reliable and effective biosimilar therapies.

### 3.4. Innovations in the Field of Bioprocessing

In the fight to accelerate biosimilar production, bioprocessing innovations have driven the development of modular and flexible manufacturing systems. Single-use bioreactor systems significantly reduce the cleaning and validation time while mitigating cross-contamination risks, thereby improving overall product safety. These conditions are necessary to work with antibodies and recombinant proteins, which are inputs into the formulation of a biosimilar [139]. In this way, these systems allow rapid adaptation to diverse production requirements, aligning with the dynamic needs of the biosimilars market [37]. Furthermore, modular designs, exemplified by advances in 3D printed microfluidic systems, enhance process versatility, allowing small-scale prototyping to be rapidly transformed into low-cost, scalable production, which is an attractive feature of bioreactors [140]. Three-dimensional printed microfluidic systems in bioprocessing have significant advantages, such as precise control over fluid dynamics and the ability to monitor biochemical reactions in real time [141]. These systems enable the integration of multiple processes in a miniaturized format, leading to reduced reagent consumption and accelerated experimentation for clone selection and media optimization. The flexibility of design contributes to advanced lab-on-a-chip applications, making them ideal for high-throughput bioprocessing [142].

The integration of PAT and real-time analytics into upstream and downstream processing enhances control and consistency in bioprocessing. These approaches facilitate continuous monitoring of critical parameters such as pH and metabolite concentrations, allowing dynamic adjustments to maintain production quality and compliance with the QbD principles [143]. Furthermore, advanced chromatography techniques, such as simulated moving bed (SMB) chromatography, significantly improve the purification of biosimilars by improving selectivity and throughput, effectively addressing challenges related to product purity and consistency [144].

## 4. Biosimilar Formulations Based on Monoclonal Antibodies and Recombinant Proteins

The formulation of biosimilars based on monoclonal antibodies and recombinant proteins is a complex process that directly determines their clinical utility by ensuring stability during storage, transport, and administration [145,146,147,148]. Precise formulation strategies, such as the use of tailored excipients and advanced stabilization techniques, not only protect protein conformation but also mitigate degradation pathways induced by physical and chemical stress [149,150,151]. An approach such as ensilication and chitosan-coated stabilizers significantly improves protein robustness, while innovative solid formulation methods offer alternatives to conventional lyophilization [152,153,154].

Chitosan-coated stabilizers have garnered significant attention in the formulation of biosimilars because of their multifaceted role in enhancing protein stability. Chitosan, a biocompatible and biodegradable polysaccharide, effectively forms a protective coating around proteins [155,156]. This coating acts as a barrier against various adverse conditions, such as aggregation and enzymatic degradation, thus maintaining the structural integrity and bioactivity of therapeutic proteins under different environmental stresses [157]. The positive charge of chitosan favors electrostatic interactions with negatively charged protein surfaces, further enhancing colloidal stability and solubility, which are critical parameters in the stability of protein formulations [158].

Furthermore, chitosan coatings can be used in nanoformulations or as excipient matrices, which are essential to extend the shelf life of biosimilars and reduce dependence on cold chain logistics [159]. The incorporation of chitosan has been reported to delay decay processes, which is vital in maintaining the quality of sensitive biological products during storage [160]. Its antimicrobial properties provide additional protection against microbial colonization, which further contributes to the stabilization of these formulations [161].

When stability technologies are explored, ensilication and lyophilization present distinct advantages and challenges. Ensilication, which involves the encapsulation of proteins within a silica matrix, protects their tertiary structures from thermal and chemical degradation, offering the potential for effective preservation at room temperature [162]. However, disadvantages include potential challenges with the complete release and recovery of protein activity postencapsulation. In contrast, lyophilization remains a well-validated technique widely accepted for protein drugs because of its ability to remove water under low pressure. This method yields a solid, stable form that retains structural integrity during long-term storage [162]. However, careful excipient design is required to minimize denaturation risks upon reconstitution [163].

The storage behavior of biosimilars is critical as even minor degradation events (e.g., oxidation, deamidation, aggregation) can significantly influence immunogenicity, potency, and pharmacokinetics [164]. To ensure product efficacy, it is imperative to conduct stability studies under the conditions recommended by the International Council for Harmonization (ICH), thus validating shelf life, transportation stability, and usability by patients. Therefore, formulation strategies must be rigorously designed to guarantee consistent quality from production to administration [165].

On the other hand, the storage behavior of high-concentration monoclonal antibodies highlights the importance of maintaining appropriate physicochemical parameters to preserve efficacy and safety [166].

Currently, numerous biosimilars derived from recombinant therapeutic proteins have emerged. Table 4 shows some of these biosimilars, with their main characteristics, which are crucial to increase competitiveness in the pharmaceutical market and improve access to quality treatments for low-income patients.

### Dissemination of Novel Ormulations

The dissemination of novel formulations is a very important element in the advancement of the safety and efficacy of biosimilars:

- Lipid encapsulation: This technology is being explored to improve the bioavailability and stability of therapeutic proteins, particularly in liquid formulations [176].
- Use of specifically designed excipients: Specific excipients are now being used to improve protein stability under adverse conditions, potentially leading to lower aggregation rates and better patient outcomes [177,178].
- Advances in nanotechnology: The use of nanocarriers offers promising strategies to deliver biologics while minimizing immunogenic responses and improving therapeutic effects through targeted delivery systems [179].

## 5. Advances in Biosimilar Formulation Technologies (Buffer-Free Strategies) and Excipients

### 5.1. Formulation and Selection of Excipients

Excipient formulation and selection challenges have received particular attention due to their associated risks with respect to protein stability and immunogenicity [3,6,180]. It is well established that high protein concentration formulations can cause aggregation, which can trigger adverse immune responses [178,181]. Consequently, various strategies are used to evaluate excipients and optimize formulations prior to approval [103,182,183,184]. The challenges of formulation extend to the development phase, where the assessment of excipient compatibility becomes paramount, as slight variations in these can alter the product and have significant clinical implications [37,185]. Research has shown that certain impurities can also lead to altered stability profiles and a rise or fall in immunogenic responses [103,182,183].

### 5.2. Buffer-Free High-Concentration Formulations and the Role of Excipients in Immunogenicity Mitigation

The development of buffer-free formulations for high-concentration biosimilars offers significant improvements in usability, particularly for subcutaneous administration. These formulations improve patient tolerability by exploiting the intrinsic buffering capacity of the protein and reducing injection site discomfort, as supported by studies that demonstrate an improved patient experience and reduced pain levels with citrate-free formulations [89]. The absence of buffer salts can mitigate the risks associated with pH instability and compatibility with device components, particularly in the context of cold chain storage [186].

Furthermore, these formulations allow for higher protein concentrations without compromising viscosity, facilitating lower injection volumes suitable for self-administration [187]. This factor is critical in improving adherence to treatment in patients, as home-based administration reduces healthcare burdens and promotes patient independence [188]. In general, these advancements underscore the potential of buffer-free biosimilars to improve patient experiences and optimize therapeutic outcomes in chronic disease management.

Innovations in excipients play a crucial role in reducing the risk of adverse reactions associated with biologic therapies. Excipients play a vital role in controlling immunogenicity during biosimilar formulation. They are selected not only for their stabilizing functions but also for their low immunogenic potential. For example, surfactants such as polysorbate 80 help prevent aggregation, while sugars (e.g., trehalose) and amino acids (e.g., histidine, arginine) stabilize tertiary structures and reduce denaturation. Replacement of citrate with polysorbate-based surfactants can prevent protein aggregation, mitigating the potential for immune responses, and improving stability [189]. Other excipients, such as trehalose, have also been used to stabilize proteins and minimize degradation, further ensuring patient comfort and safety [5]. By continuously evolving formulation strategies and improving the tolerability of biosimilars, the healthcare community can significantly enhance patient experiences and outcomes. By minimizing degradation, aggregation, and oxidation pathways, excipients help ensure consistent CQAs and reduce the likelihood of immune responses. Regulatory guidelines emphasize excipient compatibility and historical safety data, underscoring their central role in safe, patient-centered biosimilar product development.

### 5.3. Trends in Change: Buffer-Free Formulations

The formulation of biologics has significantly advanced toward unbuffered or ‘self-buffering’ formulations for high-protein drugs. Unbuffered formulations have emerged as a key trend in biosimilar design aimed at mitigating immunogenicity and improving patient safety. Traditional buffer systems, while essential for maintaining protein pH levels and stability, can inadvertently induce adverse immune reactions due to protein-buffer interactions [177]. Transitioning to citrate-free or other low-buffer systems reduces these risks, while potentially improving patient comfort during administration [184,189]. The removal of citrate buffers in high-concentration biosimilars has been associated with improved patient compliance, particularly in subcutaneous administration. Citrate buffers can cause increased pain and discomfort at the injection site due to their ionic properties, which can cause a burning sensation after injection [190]. By substituting citrate with alternative buffers such as acetate or histidine, which have been shown to minimize injection site pain, manufacturers improve the overall tolerability of the biosimilar, facilitating patient self-administration and adherence to their treatment regimens [191]. Such adjustments are especially beneficial in the management of chronic conditions, often treated with long-term biologic therapies, such as autoimmune disorders and cancers, where long-term patient adherence is crucial to treatment outcomes [5].

Recent progress indicates that high protein concentrations (>50–100 mg/mL) can offer inherent pH stability, reducing dependence on traditional buffers [192,193,194]. This phenomenon is responsible for minimizing aggregation and opalescence, as evidenced by the improved stability of high-concentration protein products from spray cooling [33,195]. Furthermore, structural analysis using techniques such as small-angle X-ray scattering (SAX) has provided information on protein–protein interactions in concentrated formulations, supporting the feasibility of eliminating conventional buffering agents while forgoing stable product stability for a robust product [196].

Recent advances in protein engineering and excipient selection have enabled biosimilars to be predominantly formulated as ready-to-use liquid solutions, thus avoiding the reconstitution challenges of lyophilized products [185]. A review of 46 high-concentration antibody products revealed that 41 products are liquid formulations, a result attributed to the use of effective stabilizers such as sugars, polyols, and amino acids that mitigate protein aggregation during storage [192,193,197]. Furthermore, a structure-function approach to amphiphilic excipients has elucidated the mechanisms by which excipient-protein interactions can be optimized to enhance the stability of the solution [194]. Alternative excipients also play a decisive role in reducing degradation pathways by minimizing interfacial tensions and protein–protein interactions, thereby enhancing the stability of high-concentration formulations [33,196].

High-concentration formulations are an innovation in themselves, allowing for alternative administration routes, facilitating subcutaneous injection of high doses but in small volumes, and minimizing dependence on infusions [96,198]. As protein concentrations increase, the use of specific excipients, such as amino acids (e.g., histidine), sugars, and surfactants, is required to maintain both solubility and adequate viscosity, avoiding aggregation problems and high viscosity levels [199,200]. Furthermore, in certain cases, the high concentration itself can provide sufficient buffering capacity to dispense with some buffers, allowing citrate-free formulations. This high-concentration monoclonal antibody (mAb) formulations face significant physicochemical challenges, particularly increased viscosity and protein aggregation [198]. Elevated concentrations lead to exponential increases in viscosity, especially near the protein isoelectric point, complicating the manufacturing and syringeability for subcutaneous administration [201]. Such viscosity can also hinder the accuracy of the dose and patient comfort [199,200]. The propensity for protein aggregation, intensified by mechanical stress and thermal fluctuations, can decrease bioactivity and increase immunogenicity, thus affecting pharmacokinetics [192].

To mitigate these challenges, strategies such as optimizing pH and ionic strength, employing stabilizing excipients such as polysorbates or amino acids (arginine, histidine), and utilizing advanced formulation techniques, including lyophilization, are essential [187]. Furthermore, predictive modeling and analytical advancements now allow a better identification of regions prone to aggregation within protein structures, facilitating informed excipient selection [202]. As the industry moves toward higher concentrations (>100 mg/mL), understanding these dynamics becomes crucial for the development of effective therapeutics [192,193,194,198].

### 5.4. Classification and Safety Profiles of Approved Biosimilar Formulations

Approved biosimilar formulations generally comprise the same functional categories of excipients as their reference products, chosen to maintain protein stability and compatibility [3]. Excipients can be classified by their function as buffers to control pH (such as phosphate, histidine, or acetate), creating an optimal environment for protein integrity [148,203]. Stabilizers, such as sugars/polyols (sucrose, trehalose, and mannitol), work by replacing water molecules and mitigating aggregation, which favors protein integrity during processing and storage [148]. Surfactants, particularly polysorbate 20 or 80, which prevent surface adsorption and subsequent aggregation, which is critical for the performance of biosimilars [7]. Amino acids as stabilizers or tonicity agents (e.g., glycine, arginine, proline), chelators such as EDTA, which are antioxidant agents and contribute to the stabilization of the overall formulation by influencing the kinetics of protein unfolding and the chemical degradation pathways and contribute to the binding of metal ions, antioxidants (methionine), and tonicity modifiers such as salts [148]. High-throughput analytical approaches further validate these strategies by allowing systematic analysis of excipient compatibility and stability, thus optimizing formulation development [7,187].

Recent studies offer a broader range of insights into the roles of excipients in biosimilar formulations. Sucrose and glycerol have been shown to improve both the thermal and conformational stability of recombinant spike proteins, illustrating the critical role of sugars and polyols in formulations [204]. Solvent systems involving tert-butanol require careful optimization to maintain protein integrity, although the complexity of interactions between different components must be considered [205]. On the other hand, novel lyoprotectants, such as sweet corn phytoglycogen dendrimers, can improve protein stability during lyophilization, acting as cryoprotectants in these processes [206]. Furthermore, a very important aspect is related to the importance of monitoring polysorbate degradation in biopharmaceutical formulations, due to its importance as a surfactant [207]. Finally, lyophilized protein formulations, prioritizing patient-centered dose design, link the choice of excipient directly to the patient’s needs [208]. Table 5 illustrates the different classes of excipients commonly used in biological formulations and their functions [193].

Most biosimilars authorized to date match or intentionally simplify the formulation of the reference product to avoid the introduction of new safety variables [209].

Filgrastim biosimilars (G-CSF) use an acetate or phosphate buffer with sorbitol and polysorbate 80, very similar to the original Neupogen^®^ formulation, which promotes protein stability and reduces the risk of immunogenicity [210,211,212,213]. Likewise, biosimilars to alfa epoetin adopt similar phosphate buffer systems and the same polysorbate stabilizing system (having removed serum albumin, as reference product, after some initial safety concerns [178]. Monoclonal antibody (mAb) biosimilars, including infliximab and rituximab, often contain the same excipients and pH as their parent compounds to ensure comparable stability and tolerability in infusion [180]. This strategic alignment of excipient profiles has been crucial to achieving clinical safety profiles similar to reference biologics, reinforcing regulatory confidence in their use [209].

Buffers deserve special attention because, while phosphate and citrate buffers are effective and have been widely used, citrate has been associated with pain and burning sensations at the injection site [214]. Citrate, a common acidic buffer, has been shown to activate acid-sensitive ion channels and induce pain after subcutaneous administration [215]. Clinical studies have shown that citrate-free and even phosphate- and glutamate-free formulations of adalimumab and other mAbs are associated with a statistically significant reduction in injection site discomfort [172,216,217]. Advances in formulation strategies have led to the successful development and approval of buffer-free monoclonal antibody (mAb) biosimilars that improve patient comfort while maintaining regulatory compliance [177,191]. A key example is the citrate-free formulation of adalimumab biosimilars such as Amjevita^®^ (Amgen), Hadlima^®^ (Samsung Bioepis), and Yuflyma^®^ (Celltrion), which have received regulatory approval both in the United States and Europe [150]. These high-concentration, buffer-free formulations are associated with reduced injection site pain and increased patient adherence [216,217]. Similarly, tocilizumab biosimilars such as Tofidence^®^ (Bio-Thera Solutions/Amgen) have introduced subcutaneous presentations with simplified buffer compositions, aligning with patient-centric design without compromising comparability standards [151]. Additionally, several trastuzumab biosimilars, including Ogivri^®^ and Herzuma^®^, have employed formulations that minimize or eliminate buffers to improve tolerability and stability [152]. These products demonstrate that buffer-free innovations are not only technically feasible but are being successfully approved within stringent regulatory comparability frameworks, demonstrating bioequivalence and improved patient-reported outcomes in clinical trials [25,32]. These examples illustrate how formulation adjustments, within the limits of biosimilar comparability, can offer tangible clinical benefits [20,36].

Similarly, the amino acid L-glutamate, used in an IL-17 antibody product as a stabilizer, has been associated with injection site reactions and is generally avoided in newer formulations [218]. In addition to local tolerability, most biosimilar excipients (e.g., sugars, polysorbates) have excellent systemic safety profiles at the doses present in injections [219,220]. Virtually all excipients in approved biosimilars are ‘Generally Considered Safe’ (GRAS) substances that have been used for decades in biologics (e.g., saline, citrate, histidine, glycine, polysorbate 80, etc.) [177,221]. Manufacturers also ensure that the quality of excipients (e.g., low levels of peroxides in polysorbates to prevent protein oxidation) mitigates any risk of adverse interactions during shelf life to ensure comparability with reference products [3].

### 5.5. Formulation Trends and Considerations on Stability, Bioavailability, and Immunogenicity

Current biologic formulations, including biosimilars, leverage refined insights into protein chemistry to improve stability, bioavailability, and reduce immunogenicity [222,223]. By carefully adjusting pH, ionic strength, and excipient selection, formulators maintain the native conformation of proteins over time. Most monoclonal antibodies (mAbs) are buffered between pH 5.0 and 6.5 to prevent aggregation near their isoelectric point and limit chemical degradation at elevated pH levels [224,225]. Excipients such as hydroxypropyl-β-cyclodextrin have been used to mitigate interfacial stress and inhibit aggregation, further stabilizing high-concentration liquid formulations [226]. This integrated approach, which considers both molecular interactions and colloidal stress factors, is critical for developing robust biologics and biosimilar formulations with improved shelf life and reduced immunogenic risk [222,223].

Modern formulations employ excipients such as sucrose or trehalose to replace water molecules around proteins, thus stabilizing tertiary structures and reducing the risk of denaturation [227]. Furthermore, surfactants such as polysorbate 80 are incorporated to inhibit surface-induced denaturation by establishing a protective layer over protein interfaces, which is crucial during transportation and handling [228]. These combined strategies not only improve protein stability but also extend the shelf life of biosimilars to 24–36 months under refrigeration, ensuring performance equivalence with reference products [227,229]. Formulations designed to withstand brief ambient temperature excursions support a robust supply chain and improve patient adherence by mitigating cold chain disruptions, reflecting advances in excipient selection and protein chemistry [228,230].

Subcutaneous formulation strategies are increasingly focusing on the adjustment of tonicity to improve the bioavailability of biologics [231,232,233]. Increasing the tonicity of the formulation, within physiologically acceptable limits, creates an osmotic gradient that can accelerate the absorption rate of macromolecules [177,208]. This approach minimizes the volume of formulation while optimizing viscosity and tonicity, thereby ensuring that a greater proportion of the dose administered reaches the systemic circulation at an appropriate rate [208].

The formulation of antibody products under approximately isotonic conditions (~300 mOsm/kg) with injection volumes of 1 mL is critical because increased osmolality or volume can lead to local pain and tissue pressure, which could reduce bioavailability [177,234]. To overcome these limitations, innovations such as coformulation of recombinant human hyaluronidase (e.g., in trastuzumab-hyaluronidase for subcutaneous use) have been implemented to transiently increase tissue permeability, facilitating administration of larger volumes while maintaining tolerability [234,235]. Modeling studies support the incorporation of recombinant human hyaluronidase, which minimizes resistance in the subcutaneous space, ensuring effective drug dispersion and absorption [236]. Consequently, while biosimilar products generally reflect the formulation approaches established by the originators, they will benefit from these advances once intellectual property restrictions are resolved [234,235].

Immunogenicity is the main safety concern for biologics, and formulation changes are carefully evaluated to determine how they can affect immune responses. A prominent case of poor immune response was the Eprex^®^ incident in the early 2000s, in which a minor formulation triggered severe antibody-mediated pure red cell aplasia [222]. In this case, the substitution of polysorbate 80 and glycine for human albumin, along with the contamination of the leachates from the syringe materials, caused protein aggregation that broke immune tolerance [237]. These aggregations are critical determinants of the immunogenic response, requiring biosimilar developers to rigorously select excipients and packaging components that minimize aggregation and the potential introduction of impurities [223].

Current epoetin alfa biosimilars have been designed to minimize immunogenicity concerns by incorporating formulation strategies that reduce aggregation, particle formation, and degradation relative to their reference products [238]. Rigorous comparative analytical studies are conducted during development and complemented by immunogenicity clinical trials before regulatory approval to confirm that these biosimilars do not exhibit increased antidrug antibody formation or immune-related adverse events [239,240]. Furthermore, the accumulated pharmacovigilance data for more than 15 years in Europe, an environment in which biosimilars have been widely used since 2006, consistently demonstrate comparable safety profiles between biosimilars and their originators, reinforcing their clinical equivalence in immunogenicity [241]. These findings support the continued clinical adoption of biosimilars of epoetin alfa as effective and safe therapeutic alternatives to reference biologics.

Improving tolerance to injection discomfort is noted as one of the main discontinuation factors for anti-TNF therapies, demonstrating the importance of formulation in maintaining constant therapeutic exposure [214,242]. Invariable dosing is critical to maintain disease control and reduce the risk of antidrug antibody development, as intermittent discontinuation of treatment can lead to decreased efficacy and increased antibody formation [243]. A real-life analysis showed higher discontinuation of treatment among patients who switched from citrate-free adalimumab to citrate-free adalimumab, reinforcing the relationship between formulation characteristics, patient experience, and clinical outcomes [191,214]. Therefore, formulation innovations play a pivotal role beyond physicochemical stability, directly influencing patient adherence and the risk of immunogenicity.

## 6. Importance of the FDA and EMA Regulatory Frameworks in the Development of Biosimilars

The regulatory framework approach presents the institutional and technical basis on which the requirements for the development, approval, and marketing of biosimilars are defined [1]. This approach, descriptive, establishes the rules for how the quality, safety, and efficacy of these are evaluated. In this course, we focus on two leading entities in biosimilar-related regulations, namely the Food and Drug Administration (FDA). Administration (FDA) and the European Medicines Agency (EMA). The FDA is responsible in the United States for protecting public health by ensuring the safety, efficacy, and security of human and veterinary medicines, as well as biological products, medical devices, the food supply, cosmetics, and products that emit radiation. The EMA is responsible for regulating only human and veterinary medicines and is involved in medical device evaluation processes within the European Union.

The emergence of FDA-approved biosimilars, particularly derived from recombinant proteins, has progressed significantly since the first approval in 2015 of Zarxio (a filgrastim biosimilar). The increasing number of biosimilars, including those for etanercept, trastuzumab, adalimumab, bevacizumab, epoetin alfa, and pegfilgrastim, has been associated with improvements in manufacturing consistency, analytical and clinical similarity to reference products, as well as economic advantages in healthcare contexts. Some studies indicate significant savings in healthcare costs and improved access to essential therapies for patients receiving these biologics [244]. Current strategies for developing biosimilars emphasize robust formulation methods, controlled expression systems, and comprehensive pharmacokinetic and pharmacodynamic evaluations to ensure comparability with original biologics, which is crucial for regulatory approval [66].

### 6.1. FDA and EMA Regulatory Approach to Biosimilars

Regulatory authorities recognize that the formulation of a biosimilar may not be identical to that of its reference product and provide guidance on what differences are acceptable or not [3]. FDA and EMA agencies require that any differences in the formulation be justified with evidence that the biosimilar does not affect safety or efficacy in terms of immunogenicity [4]. From a purely regulatory perspective, both agencies emphasize the need for a detailed characterization of biosimilar formulations to ensure that they maintain the quality standards required for regulatory approval [245,246]. This includes not only the chemical composition but also the evaluation of the physicochemical properties that influence the behavior of the drug. Regulatory bodies continue to evaluate the stability, bioavailability, and immunogenicity of these innovations, focusing on manufacturing processes that can affect the quality of the final product [5,21,247].

Biosimilar regulatory authorities consider the need for comparative immunogenicity studies during the biosimilar development process. The FDA guidance emphasizes the importance of comparative studies with reference products to assess potential immunogenicity and clinical responses prior to biosimilar approval and frames it as standard practice for the development of biosimilars [248]. For its part, the EMA advocates for equally exhaustive analyses to keep any immunogenic response within acceptable limits compared to the original formulation [249]. In this regard, the FDA also requires that biosimilars do not show clinically significant differences compared to their reference products in terms of safety and efficacy, but in this case, using a tiered framework that begins with analytical characterizations and culminates in clinical trials [250]. A crucial component of the biosimilar approval strategy of the FDA and EMA is the ‘totality of evidence’ approach. This framework evaluates cumulative data that demonstrate biosimilarity in various parameters, including analytical and functional assays, nonclinical toxicity, immunogenicity assessments, and clinical trials [251,252]. By focusing on a comprehensive dataset, regulators can interpret subtle molecular differences between biosimilars and their reference products without requiring identicality at the molecular level. In particular, the emphasis remains on identifying clinically significant differences in safety and efficacy rather than necessitating exact molecular replication [61]. This flexible, yet rigorous framework aims to increase confidence in the quality and effectiveness of biosimilars, ensuring that they meet high benchmarks comparable to reference products.

Substantial empirical evidence, sourced from comparative studies, including randomized controlled trials and real-world data, supports the safety and efficacy of biosimilars relative to their reference biologics. Research does not show significant differences in immunogenicity or clinical outcomes when switching from originator to biosimilar therapies in chronic inflammatory diseases [253]. Such findings reassure healthcare providers about the integration of biosimilars into clinical practice, fostering a wider acceptance among professionals and patients [254]. Consequently, the TOE strategy not only facilitates the scientific evaluation of biosimilars but also helps them be incorporated into treatment protocols, benefiting patient care and healthcare sustainability through cost reductions. In this way, the FDA promotes a ‘totality of evidence’ approach, which considers all available data, from analytical to clinical trials, to substantiate claims of biosimilarity [244]. The EMA, for its part, adheres to comparable principles with a strong focus on exercises that assess structural, physicochemical, and biological [188]. This exhaustive review process involves evaluating multiple batches of the biosimilar in conjunction with its reference products to verify its consistency and quality.

While these regulatory perspectives share similarities, there are subtle differences in their strategies. In particular, the FDA has allowed exemption from clinical efficacy studies for certain biosimilars, while the EMA generally favors a case-by-case analysis that often includes clinical trials [255]. This divergence illustrates the dynamic nature of biosimilar regulation and the ongoing discussions among global regulators aimed at harmonizing these processes.

A good example of biosimilar regulations can be seen in the bioequivalence trial of a rupatadine fumarate biosimilar [256], which highlights the need to harmonize formulation strategies with regulatory requirements to ensure therapeutic equivalence. Innovation in biosimilar formulation, such as altering excipients, adopting buffer-free systems, or increasing protein concentration, is allowed under current regulatory guidelines, but only within the limits established by the comparability requirements. These requirements ensure that any modifications do not result in clinically significant differences from the reference product. Regulatory agencies assess biosimilarity based primarily on analytical and functional comparability, with clinical testing being minimized but still required if residual uncertainty remains.

When modification of formulation parameters occurs in biosimilar products, additional clinical tests are often required to ensure that safety, efficacy, and immunogenicity are not adversely affected. Such modifications can include alterations in buffer systems, excipients, or concentrations, necessitating comparative pharmacokinetic/pharmacodynamic (PK/PD) trials or confirmatory efficacy and safety studies. These actions follow a risk-based evaluation that uses analytical comparability data and functional evaluations to determine whether further clinical testing is necessary [66,257].

EMA and FDA employ a ‘totality of evidence’ approach to evaluate biosimilars, but exhibit notable differences in their regulatory frameworks. EMA often requires more extensive confirmatory clinical trials, particularly for biosimilars that function through complex mechanisms or possess narrow therapeutic indices. This regulatory approach ensures that modifications to the formulation do not compromise the original therapeutic intent of the drug [258]. In contrast, the FDA has increasingly accepted robust analytical and functional data, sometimes excluding certain clinical efficacy studies if high similarity is demonstrated between a biosimilar and its reference product [117].

Furthermore, a significant regulatory distinction lies in the FDA’s unique categorization of biosimilars, allowing for a designation of interchangeability. This designation allows pharmacists to substitute biosimilars for the corresponding reference products without requiring specific prescriber approval, a feature not established similarly within the EMA regulations, where interchangeability decisions are made with individual member states [259]. Therefore, while both agencies assess the safety and efficacy of biosimilars, the implications resulting from market access and development timelines differ considerably [75].

Analyzing the evolution of regulatory practices suggests that the FDA has progressively allowed greater reliance on analytic data, reflecting increased confidence in biosimilar technologies. This trend is illustrated by FDA shifts toward less stringent clinical requirements for biosimilars that demonstrate substantial equivalence through rigorous quality assessments [260]. On the contrary, the more conservative stance of the EMA underscores the importance of clinical trials, which can prolong the time to market of a biosimilar, potentially impacting overall healthcare costs and availability [261].

### 6.2. ICH-Guided Analytical Characterization in Biosimilar Development

The development of biotherapeutic products is governed by rigorous guidelines and specifications to ensure their safety, efficacy, and comparability. The International Council for Harmonization (ICH) has established several guidelines, notably ICH Q5A, Q5B, and Q5D, which describe the steps required for the characterization of these products [262]. These guidelines emphasize the importance of appropriate analytical methodology, product characterization, and regulatory considerations for biotechnology-derived drugs, including biosimilars.

For monoclonal antibody (mAb)-rich biotherapeutic products, special emphasis is placed on the process and analytical characterization. In this regard, the importance of stress testing biotherapeutic products is highlighted to assess their stability, biosimilarity, and degradation pathways, which can significantly influence product quality [263]. Due to this, Quality-by-Design frameworks are critical to guide the development and optimization of analytical techniques for biopharmaceuticals, according to the ICH Q2 guidelines [264]. The characterization methods recommended by ICH Q6B include various analytical procedures, such as liquid chromatography and mass spectrometry, which are crucial for assessing product quality attributes (PQAs). Importantly, the need for multilevel characterization of antibody-based therapeutics is highlighted, indicating how mass spectrometry can identify different structural attributes at various levels of resolution [265]. Furthermore, the potential for charge heterogeneity to affect mAb stability and activity highlights the need for comprehensive analytical approaches, a perspective supported by studies that highlight the importance of characterization of charge heterogeneity to ensure mAb quality and efficacy [266,267].

Additionally, the biotechnology and biopharmaceutical sectors are turning to innovative technologies, such as high-throughput analytics and automated systems, to ensure rigorous quality control. Along these lines, the incorporation of advanced mass spectrometry platforms enables a faster and more accurate analysis of biotherapeutics, ensuring compliance with strict regulatory guidelines for biosimilars [268,269]. These advances in analytical methodologies align with the overall objectives of the ICH guidelines, which advocate for the integrity and scientifically sound verification of biotherapeutic products.

Likewise, the dynamic regulatory landscape, based on frameworks such as the World Health Organization guidelines and the specifics of the FDA and EMA, suggests a structured approach to biosimilar development. However, in this regard, the complexities involved in demonstrating comparability and quality control in new biotherapeutics must be highlighted, reaffirming the need for comprehensive characterization [270]. As biotherapeutic modalities diversify, innovative bioanalytical strategies become crucial to establishing the safety and efficacy of these products.

As such, any innovative formulation must be scientifically justified and supported by data demonstrating that the safety, purity, and potency of the biosimilar remain unchanged. This creates a dynamic balance: developers are encouraged to pursue improvements, but must align them with strict comparability thresholds to ensure regulatory acceptance. The results confirm pharmacokinetic bioequivalence and similar safety profiles between both formulations. This study exemplifies the practical application of formulation strategies to achieve biosimilarity, highlighting the importance of rigorous clinical evaluation in biosimilar approval. Such studies validate the effectiveness of formulation decisions in achieving biosimilarity, reinforcing the crucial role of clinical evaluation in the biosimilar development process.

Table 6 shows the most distinctive and comparative regulatory approaches to biosimilar formulations from both the FDA and the EMA.

## 7. IP Challenges Associated with the Development of Biosimilar Formulations

There is a very close relationship between regulations and intellectual property when it comes to biosimilars. Although regulatory agencies allow for differences in biosimilar formulations, biosimilar developers must navigate the scope of IP related to the active ingredients of the reference product formulation.

Biopharmaceutical molecule inventions are protected by a comprehensive set of IP protections, including product, process, and formulation patents, in order to protect both the inventor and the molecular entity and its manufacturing processes for a period of generally 20 years [276]. These patents cover the composition and therapeutic use of the original biopharmaceutical molecules and extend to innovative production techniques, such as purification and analytical characterization [277]. This protection grants inventors exclusive market rights until expiration, which, for many, incentivizes high R&D investment and encourages continued innovation, while for others, it is the opposite [278].

Following patent expiration, biosimilar manufacturers can reference these well-documented innovations while navigating the complex landscape of technology transfer and regulatory challenges, often through strategic licensing agreements [279]. Biosimilar developers must navigate a complex patent landscape that encompasses not only active therapeutic proteins but also the methodologies associated with the manufacturing processes and analytical methods used for formulation, purification, and analytical characterization [7,280]. The challenges posed by these IP strategies in the biopharmaceutical industry often require multidisciplinary collaboration between legal, scientific, and process development teams [281]. Such collaborations aim to develop robust quality and manufacturing protocols that withstand legal scrutiny while ensuring equivalence with the reference product. IP in the biosimilar space is particularly complex due to the layered nature of biopharmaceutical patents, which can cover various aspects of a product, such as molecular structure, manufacturing techniques, and analytical methods [277]. Regulatory authorities typically require a demonstration of biosimilarity regardless of these complexities, forcing developers to provide compelling evidence that manufacturing variations do not result in meaningful differences. This environment has driven innovations such as the use of novel orthogonal analytical platforms that offer new foundations for IP protection based on increased sensitivity and precision [37]. These innovations not only drive market differentiation but also provide a competitive advantage in the increasingly crowded biosimilar market.

In many cases, strategic alliances and licensing agreements have become essential mechanisms to overcome these obstacles, allowing access to complementary technology and a shared regulatory vision [282]. These alliances not only mitigate the risk of costly litigation but also facilitate market entry by taking advantage of the capabilities of partners in innovation and regulatory compliance [33].

As new biosimilar formulation strategies are developed, so do the IP challenges associated with this evolution [283]. Biosimilar differentiation is largely dependent on formulation innovations, but many existing patents cover a wide range of excipients and stabilization techniques that make them difficult to use in biosimilars [35,185]. In this regard, original inventors can apply for patents on innovative excipients or novel delivery mechanisms to protect investments in developing unique formulations [178,284,285,286].

However, there are other challenges in patent protection for original biologics, which in some cases extend beyond their 20-year expiration, trying to maintain market exclusivity by continuously filing patent applications for new indications, formulations, or methods of administration [287]. Companies may employ strategies such as patent stacking, which complicates market entry strategies for biosimilars. The balance between launching innovative formulations and navigating this landscape requires astute legal strategies coupled with sound scientific development [288]. A “patent thicket” refers to the dense, overlapping patent portfolio that a pharmaceutical company accumulates around a single product, encompassing not only the primary active ingredient but also numerous ancillary patents covering formulation techniques, manufacturing processes, delivery devices, and specific methods of use [19]. This strategy creates a legally complex environment that can effectively deter a competitor from developing biosimilars, even after the expiration of the primary patent [289]. For example, original companies intentionally apply for secondary patents on trivial modifications, such as changes in excipient combinations, which, when aggregated, form a ‘patent thicket’, making it both legally and financially difficult for competitors to design around the core innovation [290,291].

These strategies can substantially delay market entry for biosimilars. In this regard, evolving definitions of interchangeability and protocol changes in biosimilar development complicate the IP landscape by providing competitive opportunities and substantial risks for originator companies [292,293]. This patent layering creates significant barriers for biosimilar manufacturers, who must match the reference formulation and risk litigation or develop non-infringing alternative formulations that often require additional comparative studies [294]. The case of AbbVie Humira^®^ (adalimumab) illustrates this strategy, where despite the patent for the lead antibody expiring in 2016, at least 20 subsequent formulation patents have ‘extended’ product exclusivity by covering various buffer and excipient configurations [189]. This patenting strategy ensures that biosimilar competitors must design around these patents or enter licensing agreements with the original manufacturer, thus delaying their entry into the market and prolonging exclusivity periods even after the original compound is no longer protected by patent [291]. The dense network of overlapping patents not only reduces incentives for the development of new biosimilars but also complicates the legal landscape for these companies, increasing costs and diverting capital concentration from immunogenicity studies [295]. The dynamics of litigation and settlements under regulatory frameworks such as the BPCIA can force biosimilar companies to enter agreements that delay launch dates or impose licensing fees, even for marginal similarities in formulation [287,294].

Other risks for biosimilar manufacturers may include entry barriers created by large pharmaceutical conglomerates to prevent new competitors. There may also be a relationship between the original brands of a pharmaceutical product with historical intellectual property rights generated by the “loyalty” of physicians and patients to a particular brand [2]. Similarly, the arrival of high-concentration, buffer-free biosimilar formulations introduces formulation attributes that can alter traditional exclusivity contracts established in formulations of reference products already patented [190,296]. The importance of such innovations is that not only do they improve patient convenience and reduce administration volumes, but they can also challenge the scope of patent claims, as these new attributes may not have been considered in the original exclusivity agreements [192]. This divergence creates complex legal and regulatory dilemmas in which innovating companies could claim that changes in buffer concentration or composition represent substantial improvements that justify extended protection [297]. Consequently, stakeholders should reexamine exclusivity frameworks to ensure that they appropriately balance innovation incentives and the promotion of competitive biosimilar markets [296]. In general, these developments underscore the need for clearer contractual and regulatory guidance to mitigate potential disputes that arise from the evolution of biosimilar formulations. [280].

The main IP challenges and formulation strategies that biosimilar developers must address to ensure successful market entry, along with a detailed description, can be seen in Table 7. Regarding formulation, this table emphasizes the importance of taking advantage of innovation in the pharmaceutical industry to optimize the safety, stability, and patient usability of the biosimilar without infringing existing patents. This includes selecting established excipients, minimizing immunogenic risk, ensuring compatibility of key pharmaceutical attributes with the reference product (such as dosage form and route of administration), and performing extensive stability and compatibility testing. It is critical that developers engage with regulatory agencies from the outset when proposing formulation differences and align formulation design with the IP landscape to avoid late-stage legal setbacks. In general, this dual approach, which balances intellectual property risk mitigation with scientific and regulatory rigor, is critical to developing biosimilars that are not only legally viable but also clinically sound and patient-centered.

## 8. Discussion

The formulation and development of biosimilars face complex and multifaceted challenges that span the scientific, economic, and psychosocial domains. Successful biosimilar implementation depends on rigorous scientific validation of similarity, effective stakeholder communication, and collaboration across health systems. With successful implementation, biosimilars can significantly reduce healthcare costs and expand patient access to vital biologic therapies, thereby improving the management of chronic disease. To contextualize recent findings and trends in biosimilar formulation, this discussion examines their development, from innovations in formulation, design, manufacturing, and analytical advances to regulatory evolution by the FDA and EMA, as well as barriers related to intellectual property, clinical adoption factors, and the transformative impact of digital tools and AI.

### 8.1. Innovation in Formulation and Patient-Centered Strategies

Biosimilar formulation strategies are increasingly based on patient-centric considerations and innovative approaches that improve usability without compromising quality. Improving patient acceptance of biosimilars extends beyond formulation adjustments to include comprehensive patient education and transparent communication. The participation of patients in treatment decisions and the resolution of concerns about biosimilar efficacy and safety can significantly enhance their willingness to use these therapies [298]. Furthermore, educational interventions aimed at clarifying the equivalence of biosimilars to their reference products can alleviate fears, thus fostering greater acceptance [5]. The challenge of managing viscosity and protein aggregation in high-concentration formulations remains a significant limitation in biosimilar development. While these formulations are essential to reduce injection volumes and improve patient adherence, they introduce complex biophysical risks. Aggregation not only threatens structural stability but can also increase immunogenicity, undermining the clinical equivalence of the biosimilar. Elevated viscosity, on the other hand, can hinder manufacturability and patient self-administration. These issues highlight the need for an integrated approach that combines early-stage analytical characterization, rational screening of excipients, and advanced formulation technologies. As biosimilar developers seek to innovate through buffer-free or high-concentration systems, trade-offs between patient-centric delivery and molecular stability must be carefully navigated within the constraints of comparability and regulatory approval. A paradigm shift towards high-concentration, low-volume formulations (including buffer-free or self-buffering systems) has paved the way for more convenient subcutaneous administration, improving patient comfort and adherence. In this regard, biosimilar monoclonal antibodies have been designed for subcutaneous administration at higher concentrations, reducing injection frequency and volume to improve patient experience without decreasing treatment quality [297,298]. In addition, subcutaneous administration of high-concentration biosimilars represents significant progress in therapeutic modalities. This pathway improves dosing convenience by allowing for reduced administration frequency, thereby easing the logistical burden on healthcare systems and patients alike [80]. The transition from intravenous to subcutaneous administration requires careful consideration of formulation, particularly with respect to viscosity and stability [80]. High concentrations in formulations can increase viscosity, making delivery through subcutaneous administration injection challenging unless optimized with suitable excipients. Therefore, improved formulation strategies, using innovative excipients, are critical to maintaining product stability while providing patient-friendly delivery mechanisms [5].

Similarly, the use of citrate-free buffer systems in biosimilars such as adalimumab has been shown to minimize injection site pain, increasing patient satisfaction and comfort [209,299,300]. These formulation innovations, which include the introduction of new excipients and the removal of components associated with adverse reactions, seek to maintain or improve safety profiles (e.g., by reducing aggregation or immunogenicity) while optimizing ease of use. All these changes are supported by comprehensive comparability data that ensure the clinical performance of the biosimilar. Regulatory authorities require that any differences in formulations be justified with evidence of unchanged efficacy and safety, underscoring the need for extensive empirical stability, bioavailability, and immunogenicity testing during development [301]. To this end, thorough preclinical and clinical evaluations of any new formulation approach, along with post-launch immunogenicity surveillance, are recommended to confirm that patient-centered improvements do not introduce new risks. Post-launch, continuous immunogenicity monitoring is fundamental to biosimilar management. Even minor structural variabilities, resulting from processing conditions or interactions with packaging materials, can induce anti-drug antibody (ADA) responses, making diligent pharmacovigilance essential [298]. Regulatory bodies mandate these activities to protect patient safety and maintain the therapeutic effectiveness of the biosimilar, ensuring that any potential immunogenicity issues are identified and addressed promptly [5]. A robust monitoring system can also facilitate the collection of real-world data, which is necessary for adjustments in patient management strategies aimed at mitigating adverse effects and optimizing therapeutic outcomes.

Balance of innovation with biosimilarity often requires an interdisciplinary approach, with formulation scientists, clinical experts, regulatory specialists, and legal counsel working together to select optimal formulations that improve patient outcomes within the bounds of regulatory expectations and patent restrictions. This collaborative and patient-centric strategy helps position biosimilars as accessible and user-friendly alternatives to reference biologics without compromising quality or patient safety.

### 8.2. Analytical and Manufacturing Advances

Advanced analytical characterization and manufacturing innovations have become cornerstones of biosimilar development, enabling robust demonstration of similarity to reference products. High-resolution methods such as two-dimensional NMR spectroscopy are now used to compare higher-order protein structures, ensuring that the three-dimensional conformation of the biosimilar aligns with that of its reference product [296,302]. Similarly, X-ray crystallography and cryo-electron microscopy can provide information at the atomic level. These methods have confirmed the virtually identical molecular architecture of some biosimilars (e.g., infliximab) [296]. Such structural analyses, while sensitive enough to detect minor conformational divergences, are combined with functional assays (cell-based potency testing, receptor binding studies) to verify that the observed differences do not have a clinically significant impact on the bioactivity of the latter. This orthogonal analytical approach, which integrates detailed physicochemical characterization with biological function testing, constitutes a robust platform for establishing biosimilarity. In fact, extensive side-by-side comparisons, including peptide mapping, glycan profiling, aggregation analysis, and in vitro bioassays, have successfully demonstrated no significant differences between biosimilars and references. In one case, a proposed biosimilar infliximab (ABP 710) was shown to be highly similar to the originator using a variety of analytical techniques, providing evidence that met the requirements of the FDA and EMA [51]. These advances in analytical resolution and sensitivity ensure that even subtle variations in post-translational modifications or impurity profiles are detected and assessed. Techniques such as MALS, DLS, and DSC are now routinely applied to assess colloidal stability, detect aggregates, and characterize the thermal stability of biosimilars [106]. All these techniques are crucial to predict shelf life and immunogenicity risk. When high-resolution structural data are aligned with rigorous functional and stability assays, developers can confidently demonstrate that any small physicochemical differences are clinically irrelevant.

Replicating PTMs is among the most complex challenges in biosimilar development. These modifications, such as glycosylation, deamidation, and oxidation, are highly sensitive to cellular systems and manufacturing conditions, leading to potential variations that can affect pharmacokinetics, efficacy, and safety. Even small differences in glycan structures can alter receptor binding and immunogenic potential. Consequently, developers must use high-resolution analytical methods to compare PTM profiles with reference biology. Immunogenicity remains a critical concern in formulation because slight deviations in protein structure, aggregates, or host cell impurities can trigger immune responses. These may reduce therapeutic efficacy or lead to adverse clinical outcomes. Therefore, immunogenicity testing is a central pillar of regulatory comparability assessments, alongside stability and pharmaceutical equivalence.

Parallel to analytical advances, biosimilar manufacturing processes have evolved to emphasize consistency, control, and efficiency. Given the inherent biological variability in the production of complex biologics, manufacturers implement extensive process characterization and validation to ensure that each batch of biosimilars remains within strict quality specifications. QbD has been widely adopted, involving the systematic identification of critical quality attributes and process parameters, followed by the design of robust processes that consistently meet those objectives [169]. The development of new processes uses in silico modeling and simulation tools to define optimal operating ranges. In this regard, computational fluid dynamics and bioreactor modeling help optimize cell culture conditions and scale-up steps. During production, the PAT and real-time monitoring systems are integrated to achieve adaptive control of the manufacturing process. Sensors and automation continuously track critical parameters (pH, temperature, metabolite levels, product titer, etc.), providing immediate feedback that enables dynamic adjustments. This real-time monitoring, coupled with statistical process control, greatly reduces batch-to-batch variability and ensures that the biosimilar remains within the predefined design space [95]. Importantly, these manufacturing innovations also include the transition to continuous bioprocessing and the use of single-use systems, which improve operational efficiency and product consistency. Continuous manufacturing can minimize downtime and contamination risk while allowing more precise control of process conditions, resulting in a more uniform product profile [303,304]. The adoption of these technologies requires significant investment in advanced equipment and data analytics, but is driven by the need to increase throughput and cost-effectiveness without sacrificing quality. Together, the convergence of sophisticated analytical methodologies with improved manufacturing practices allows biosimilar developers to maintain strict similarity to the reference biologic in all production batches. These advances not only meet regulatory expectations for complete side-by-side comparability but also improve internal quality assurance, thus reducing development risks and ensuring that patients receive a product equal in safety and efficacy to the originator, but at a fraction of the original cost.

### 8.3. Regulatory Evolution and Market Access

Regulatory frameworks in the United States and Europe have progressively evolved to support the efficient development of biosimilars, focusing on a science-based approach of ‘totality of evidence’ to establish biosimilarity. Both the FDA and the EMA have issued guidelines highlighting the importance of thorough analytical and functional characterization, which in many cases can reduce or replace the need for large-scale clinical trials. This paradigm shift in regulatory strategy, supported by the accumulation of evidence that analytics can reliably predict clinical performance, has simplified the biosimilar approval process. For example, current guidelines allow that if a biosimilar shows high similarity using state-of-the-art analytical techniques and pharmacodynamic studies, confirmatory clinical efficacy trials can be reduced or omitted [37]. EMA also adopts rigorous comparability exercises using orthogonal analytical methods and in vitro functional testing, allowing the extrapolation of clinical data from one indication to another once biosimilarity is demonstrated. Both regulatory agencies place unprecedented weight on analytical data, reflecting a consensus that comprehensive physicochemical and bifunctional evidence can eliminate redundant clinical studies in many settings, reducing time and costs. In practical terms, this regulatory evolution accelerates market access, directly impacting patients, as biosimilars can be available earlier and at a lower development cost, resulting in lower payment per treatment, as manufacturers can focus on high-quality analytical demonstrations instead of duplicating costly efficacy trials. On the other hand, agencies have been actively promoting innovative manufacturing approaches by incorporating QbD and PAT principles into their review process, driving the industry to adopt advanced process controls and ensuring consistent product quality throughout its life cycle [169]. Advanced analytical techniques, such as LC-MS and NMR, are used to monitor structural and functional consistency over time [7]. The correlation of process data with clinical performance helps to understand the impact of process changes and informs control strategies that minimize risk. This proactive approach is central to the regulatory philosophy regarding post-approval process changes. Another notable regulatory concept is interchangeability (particularly in the US), a designation that requires additional evidence that a biosimilar produces the same clinical outcome when patients switch from one reference product to another. Interchangeability assessments impose even stricter scrutiny on any differences in formulation or administration. To date, interchangeable biosimilars (for example, certain insulin glargine and adalimumab products) have used formulations essentially identical to their reference counterparts to meet this high standard of interchangeability, highlighting regulators’ caution that substituting a biosimilar does not create new safety or efficacy issues [3]. Beyond scientific considerations, regulators and policymakers are also addressing the practicalities of market access. Initiatives are being developed for international regulatory collaboration and harmonization of guidelines to minimize discrepancies between regions, thus simplifying the global development and approval of biosimilars (e.g., efforts to align the FDA and EMA evaluation criteria) [1]. This collaborative regulatory environment reduces duplication of efforts and encourages greater entry into the market of biosimilars. However, regulatory agencies still maintain strict post-approval monitoring and pharmacovigilance requirements to ensure that the real-world use of biosimilars confirms their safety and efficacy [24]. Policy developments are a key enabler of market access, enabling healthcare systems to realize the economic and therapeutic benefits of biosimilars on a larger scale.

Regarding tolerability and patient outcomes, the evaluation and approval process for biosimilars by regulatory agencies such as the FDA and the EMA is crucial to ensuring the safety, efficacy, and tolerability of these therapeutic agents. Both agencies have established very rigorous criteria for biosimilar products, which require substantial evidence of similarity to reference biologics. Specifically, the EMA requires biosimilars to demonstrate similarity in terms of quality, efficacy, and safety, while the FDA emphasizes safety, purity, and potency criteria during the approval process [2,52,274]. These strict requirements mean that biosimilars must undergo a series of analytical, preclinical, and clinical studies to confirm their equivalence to the original biologic drug in terms of pharmacological activity and clinical outcomes [2,5].

The results of these assessments indicate that, once approved by these agencies, biosimilars are generally well tolerated by patients and maintain safety profiles similar to those of their reference products. For example, a study analyzing oncology biosimilars in Europe used the EudraVigilance database to assess safety and concluded that biosimilars are as safe and effective as their original products in all approved indications [47]. Furthermore, recent analyses indicate that switching from the original infliximab to its biosimilar does not compromise disease control in patients treated with rheumatoid arthritis (RA). PERFUSE evidence (a noninterventional study) confirmed that there were no significant safety differences between biosimilar and reference infliximab when patients transitioned from one to the other, suggesting comparable efficacy in the management of inflammation [305,306,307]. Furthermore, real-world data collected from various observational studies show that biosimilars provide therapeutic benefits like the originator and improve cost-effectiveness without sacrificing patient outcomes. For example, switching strategies have maintained or improved disease management parameters among patients with rheumatic disease, as highlighted in studies evaluating biosimilar adoption [308]. This growing body of literature supports the safety and tolerability of biosimilars, reinforcing their role as viable alternatives in the management of chronic diseases, including RA and inflammatory bowel diseases [309].

Long-term safety data for several biosimilars sold indicate that they can be used safely, with minimal adverse events reported compared to their reference counterparts [75]. For example, a biosimilar to pegfilgrastim (Stimufend) was shown to have functional characteristics and patient tolerability similar to those of its reference drug, Neulasta [52,86]. This consistency in safety and efficacy fosters greater confidence among physicians and patients in biosimilars as viable treatment options. Large-scale analyses reveal that patients experience therapeutic benefits comparable to those obtained with standard treatments, while benefiting from reduced treatment costs, which alleviates the financial burden on both healthcare systems and patients [310].

Direct evidence from regulatory evaluations further strengthens claims related to patient tolerability and outcomes [311,312,313]. The FDA’s approval of citrate-free formulations of adalimumab biosimilars, such as Amjevita and Hadlima, underscores significant advances in patient-centered care by reducing injection site pain, which is a critical factor affecting treatment adherence and outcomes [314,315]. The removal of citrate buffers in these formulations has been shown to enhance tolerability, indicating that formulation modifications can produce meaningful patient benefits without compromising the intended therapeutic impact [316].

Furthermore, the European Medicines Agency (EMA) recognizes improved tolerability in biosimilars such as Hyrimoz and Idacio, supported by pharmacokinetic studies and real-world evidence that demonstrate their efficacy without loss of therapeutic effect [5,317]. These findings align with the assertion that alterations in excipient compositions can lead to better patient adherence due to fewer adverse effects, reinforcing the role of patient-centered improvements in biopharmaceutical development [318].

### 8.4. Intellectual Property and Strategic Development

IP plays a critical role in shaping biosimilar development strategies. As noted, some original-molecule owners employ “patent thickets,” dense overlapping patent portfolios around a biologic, to maintain a form of market monopoly and hinder biosimilar entry [289,290,291]. Given this landscape, biosimilar manufacturers require strategic foresight to carefully design their products and processes to avoid infringing active patents or be prepared to challenge them legally. In some cases, the most IP-secure formulation option is to use only well-established (off-patent) excipients and components, even if the reference product contains a proprietary stabilizer or buffer. This dynamic has driven certain formulation innovations, for example, the transition to buffer-free formulations or the use of alternative excipient combinations not covered by patents, to differentiate a biosimilar from the originator without violating IP rights. Although these alternative design strategies may allow for a faster route to market, they must be executed without compromising the quality and comparability of the biosimilar. Patent litigation remains a major hurdle even after a biosimilar has received regulatory approval, where patent disputes can delay its commercial launch by months or even years [319]. To mitigate these risks, biosimilar companies are developing IP-related strategies, including early identification of potentially conflicting patents, filing challenges, or alternative technologies, and careful planning of market entry to coincide with patent expiration. The involvement of legal experts throughout development has become standard practice to ensure that biosimilar products can be developed without being mired in protracted legal battles. However, these practices increase the cost of the final product.

In the patent litigation case AbbVie Inc. v. Boehringer Ingelheim, AbbVie asserted multiple formulation and method-of-use patents related to its blockbuster drug Humira^®^ (adalimumab). The strength of the AbbVie patent portfolio poses significant barriers for competitors attempting to enter the market. Boehringer initially challenged several of these patents, but ultimately agreed to delay its entry until July 2023, suggesting the perceived robustness of AbbVie’s formulation intellectual property (IP) [289]. Patent thickets, particularly related to biologics such as Humira, complicate the market landscape for biosimilars, which require strategic navigation to circumvent or challenge existing patents [320]. The extensive protections granted to AbbVie illustrate the challenges that other manufacturers face in attempting to introduce competing products in a market characterized by rigorous patent enforcement [320]. Boehringer’s decision underscores the ongoing dialog surrounding patent law intricacies and their implications for pharmaceutical innovation and competition [319]. These examples illustrate how biosimilar developers must anticipate ‘patent thickets’ and engage in early FTO assessments to avoid infringement. Public databases such as the FDA’s Purple Book and USPTO filings, along with competitive intelligence tools, are now essential to map formulation-related IP barriers.

FTO assessments in biosimilar development are critically important, especially when considering formulation innovations such as buffer changes, high-concentration adaptations, or new excipients and high-concentration adaptations. These assessments help determine whether a biosimilar can be developed without infringing existing patents, which is vital as biologics patents expire and competition increases [321]. Conducting a comprehensive patent landscape analysis is integral to this process, which involves identifying patents related to formulations and manufacturing methods, assessing geographic scope and expiration, and evaluating legal strategies for potential noninfringement [3]. Public databases such as the FDA Purple Book and the EMA European Public Assessment Reports (EPAR), along with commercial tools such as PatentScout^®^ or Derwent Innovation^®^, are frequently used, as are advanced tools for tracking patent information [3,37]. In some cases, legal strategies such as designing around patent claims, filing IPR requests, or applying for patent licenses are applied. Furthermore, FTO considerations can help biosimilar developers minimize litigation risks, thus facilitating accelerated regulatory pathways and promoting innovation [322,323]. The FTO process typically involves a thorough analysis of the patent landscape by intellectual property specialists, including: (1) identifying active patents covering the formulation, excipients, delivery systems, and manufacturing methods of the reference product; (2) evaluating patent claims for geographic scope and expiration; and (3) determining the potential for noninfringement or invalidity.

Beyond defensive patent enforcement measures, strategic development in the field of biosimilars increasingly involves collaborative and innovative approaches. Some biosimilar manufacturers partner with academic institutions or form consortiums to advance shared analytical and manufacturing technologies [323]. These collaborations allow the pooling of knowledge and resources to address common challenges, accelerating progress in areas such as high-throughput analytics, bio-simulation, and process optimization. Joint research initiatives have contributed to industry-wide advancements in PAT tools, QbD methodologies, and other technological innovations that benefit all stakeholders [324]. This open innovation model not only helps individual developers improve their biosimilar programs but also provides regulators with more data and case studies to refine regulatory guidelines, paving the way for future biosimilars. From a market strategy perspective, companies also invest in training and outreach as part of their development. By engaging with healthcare professionals, patient advocacy groups, and insurers early on, biosimilar developers seek to build trust and understanding of their products prior to launch. This stakeholder engagement can prevent misinformation and create a more receptive market environment when the biosimilar becomes available. In essence, strategic biosimilar development now extends far beyond the laboratory and encompasses IP expertise, cross-industry collaboration, and proactive market conditioning, all crucial to successfully bringing a biosimilar to patients.

### 8.5. Clinical and Psychosocial Considerations

As biosimilars become an integral part of clinical practice, their impact and acceptance in community patients have become a tangible reality. Clinically, numerous studies in therapeutic areas (rheumatology, gastroenterology, oncology, etc.) have shown that switching patients from a reference biologic to a biosimilar does not compromise efficacy or safety. For chronic conditions such as rheumatoid arthritis or inflammatory bowel disease, for example, patients treated with biosimilars experience disease control and adverse event rates equivalent to those of the original drug [325,326]. Post-marketing surveillance data from both the FDA and the EMA continue to affirm that approved biosimilars have safety profiles consistent with their reference products. In particular, recent clinical switching studies have found no emerging safety issues or loss of therapeutic effect when patients transition to biosimilars under medical supervision, indicating that patient care can continue without restrictions from the switch [327]. These findings are crucial to strengthening the confidence of physicians and patients and supporting regulatory decisions to extrapolate indications from biosimilars. In addition, biosimilars have expanded access in costly diseases by providing equally effective therapies at lower cost, which can improve overall health outcomes through earlier or sustained treatment of conditions that were previously resource-limited with the original biologics.

Despite the excellent clinical performance of biosimilars, psychosocial factors significantly influence their acceptance and adherence to treatment. A well-documented problem is the nocebo effect, in which negative expectations or anxiety about a biosimilar can lead patients to perceive lower efficacy or increased side effects due solely to psychological factors [328,329]. Some patients, upon learning that they have switched to a biosimilar, report decreased therapeutic benefit or new symptoms, even when objective measures show that the disease remains controlled [330]. Studies have found that apprehension about biosimilars, often due to a lack of familiarity or misconceptions, can result in higher rates of discontinuation or non-compliance with treatment [331,332]. These psychosocial barriers underscore that the adoption of biosimilars is not only a scientific and regulatory endeavor, but also a human one. To address this, ongoing education and communication strategies are critical. Healthcare providers play a critical role in defining biosimilars positively and reassuring patients about their rigorous approval process and equivalence to originators. Improving patient and physician familiarity and comfort with biosimilars has been shown to reduce anxiety and improve uptake [2,253,333]. Transparent conversations about biosimilar evidence, along with supportive resources (e.g., patient education leaflets, nurse-led seminars), can anticipate concerns generated by the nocebo effect. In specialties that rely on biologic therapy, such as oncology or immunology, addressing these ‘soft’ factors is especially important, as unjustified fear of lower efficacy or new side effects can influence prescribing habits and patient consent [334,335]. Therefore, in conjunction with scientific development, manufacturers and healthcare systems are investing in research and psychosocial interventions to ensure a smooth transition to biosimilars in practice. Ongoing pharmacovigilance and real-world studies also contribute to reassurance, as the accumulation of data over years of biosimilar use helps to dispel lingering hesitancy. Clinical evidence supports the idea that biosimilars are interchangeable with their reference products in terms of patient outcomes, but achieving optimal benefits requires addressing psychological and communication aspects. Proactive education, patient participation, and physician training are essential to overcome hesitancy, thus improving adherence and maximizing the therapeutic and economic advantages of biosimilars [336,337].

### 8.6. The Role of Digital Tools and Predictive Modeling

Technological innovations, particularly digital tools, data analytics, and AI, are radically empowering biosimilar development and manufacturing, marking a hallmark of more predictive and efficient biopharmaceutical production. One of their main applications is process development and control with advanced data analytics and machine learning algorithms, which can analyze the vast datasets generated by bioreactors and analytical instruments to identify patterns or anomalies that human operators might miss. For example, AI-based models have been used to predict the impact of subtle changes in fermentation or purification parameters on critical biosimilar product quality attributes [57]. By training on historical manufacturing data, machine learning systems can flag potential process deviations in real-time and even recommend adjustments before product quality is compromised. This predictive capability enables a shift from reactive to proactive process control and a realization of smart manufacturing principles in the biologics field. In practice, the integration of PAT with real-time AI analytics enables fine-tuning and dynamic process adjustments. If a sensor detects a deviation in pH or temperature, the system can immediately compensate or alert technicians, thereby maintaining optimal conditions continuously throughout the process. Such adaptive manufacturing not only improves consistency and yield but also reduces the risk of batch failures, directly contributing to cost savings and reliability. Regulatory agencies have recognized the potential of these technologies. The FDA, for example, has encouraged the adoption of advanced manufacturing and control techniques because they can strengthen product quality assurance. Over time, fully automated production lines can be envisioned in which AI models, under regulatory oversight, manage routine decision-making to ensure that each batch of a biosimilar remains within specifications.

Silico modeling and simulation have also become invaluable in the R&D phase, accelerating the design and characterization of biosimilars [338]. Computational tools, including molecular modeling and emerging AI methods, such as deep learning (e.g., graph neural networks), make it possible to simulate the structural behavior of biologics and predict how changes might influence the stability or function of the final product before it is made. These digital approaches can complement laboratory experiments by highlighting the most promising formulation or process conditions before empirical testing. For example, deep learning models have been applied to predict protein folding outcomes or identify sites prone to aggregation, guiding formulation scientists in the selection of stabilizing excipients or process adjustments [127,339]. By accurately modeling molecular interactions, these tools help to ensure that the structural conformation and biochemical properties of the biosimilar reflect those of the reference product under various conditions. Integrating virtual simulations into development processes creates a powerful feedback loop, where hypotheses can be rapidly tested in silico, refined, and then validated in the lab, significantly improving efficiency. Indeed, an ecosystem is emerging where virtual experimentation partially replaces physical experimentation, especially in the early stages of development, where numerous candidates or endpoints are being examined. The synergy between digital environments and traditional wet lab work has been cited as a crucial factor in driving development efficiency and success rates in an increasingly competitive global biosimilars market [340,341,342,343,344,345]. Likewise, it should be noted that digital innovations are not limited to the development phase, but also extend to clinical and post-marketing environments [20,33]. In this case, AI-powered pharmacovigilance tools can analyze real-world data (electronic health records, patient registries) to detect any immunogenicity signals or adverse events with greater sensitivity and speed than traditional reporting systems [2,346]. All these digital and computational advances align with Industry 4.0 principles and are gradually transforming the biomanufacturing landscape. Using big data and predictive models, biosimilar developers can make more informed and faster decisions, reduce trial and error, and ensure a higher probability of success in both the demonstration and maintenance of biosimilarity throughout the product lifecycle [347]. As technology matures, it promises an era of smarter biosimilar development, where sophisticated algorithms and simulations are fully integrated with bench-top science to deliver safe, effective, and affordable biologic therapies more efficiently than ever before.

In terms of global access to biologic therapies, innovations in biosimilars, including cost-effective production methods and adaptable manufacturing models, can significantly lower expenses. These advances may facilitate local production capabilities, promoting greater availability in low-resource regions [63,348]. Regulatory policies that encourage the development of biosimilars also play a critical role in democratizing access to advanced therapies, resulting in substantial savings and improved patient outcomes in various healthcare settings [24].

## 9. Conclusions

The formulation of biosimilars represents a strategic axis in the development of modern biopharmaceuticals that combine technological innovation, strict regulation, and intellectual property challenges. This systematic review has identified important advances such as buffer-free formulations, high-concentration systems, and rational use of excipients, which improve stability, immunogenicity, and patient experience. Technologies such as QbD, PAT, and artificial intelligence-based tools allow for the anticipation of critical risks and the optimization of scale-up processes. From a regulatory perspective, there is increasing flexibility from agencies such as the FDA and EMA to accept innovative formulations, provided that biosimilarity with the reference product is maintained. However, barriers imposed by intellectual property continue to limit timely access to biosimilars.

One of the most important keys to biosimilar development lies in rigorous formulation design and comprehensive characterization essential to producing biosimilars that match their reference products in quality, safety, and efficacy. Developers are embracing patient-centric formulation innovations (such as high concentration, easy-to-inject formats, and excipient optimizations) that can improve adherence and convenience without deviating from the reference product profile. At the same time, tremendous progress in analytical techniques, from high-resolution structural assays to sensitive functional and stability tests, supports the robust demonstration of biosimilarity. These scientific advances, when combined with cutting-edge manufacturing practices (QbD-based process design, real-time monitoring, and even continuous production systems), ensure that biosimilars are produced with consistent quality and at scale. The constantly evolving regulatory frameworks in the US, EU, and other regions have embraced this science-based approach, prioritizing analytical and quality data over exhaustive clinical trials. Regulators have created more streamlined evidence-based approval pathways. This not only maintains high safety standards but also accelerates patient access to biosimilars and fosters a more competitive market in the pharmaceutical industry. Meanwhile, managing the intellectual property landscape remains a strategic necessity, with biosimilar sponsors needing to proactively manage patents through design-focused innovations and legal strategies to avoid or overcome patent thickets created by originator companies. Equally important are the clinical and psychosocial dimensions, as real-world experience confirms that biosimilars are as effective and safe as originators, but maximizing their impact requires building trust between prescribers and patients. Education, transparent communication, and post-marketing surveillance contribute to increasing confidence in biosimilars. The rise in digital tools, such as AI and silico modeling, is emerging as a unifying force, bridging these gaps by improving decision making and efficiency in formulation, manufacturing, and even patient monitoring. Thus, the field of biosimilars has matured rapidly, and collective efforts in formulation science, analytical rigor, regulatory policy, intellectual property strategy, patient engagement, and technological innovation have converged to make modern biosimilars a success story in pharmaceutical development. This article seeks to provide integrative guidance for the design, evaluation, and regulation of biosimilars that are safe, effective, and sustainable, promoting their adoption in global healthcare systems.

The highlight of this article offers a novel multidimensional integrative framework for biosimilar formulation by integrating pharmaceutical design, regulatory comparability, intellectual property strategy, and digital innovation, domains often addressed in isolation. Unlike conventional reviews, this article synthesizes how formulation options, such as buffer-free and high-concentration systems, can be strategically aligned with regulatory flexibility and legal constraints to accelerate biosimilar development. By constructing this work into three interdependent domains: formalism science, regulatory compliance, and intellectual property strategy, we provide a comprehensive framework rarely unified in the current literature. From emerging trends in buffer-free and high-concentration systems to the flexibility allowed by FDA and EMA biosimilar comparability standards, and the legal constraints imposed by formulation patents, each dimension of development is explored in relation to the others.

A key novelty of this work lies in its analysis of how AI, through digital twins, machine learning, and predictive models, can revolutionize the biosimilar development process. By enabling real-time monitoring, process optimization, and in silico formulation testing, AI offers unprecedented tools to reduce risk, improve bioequivalence, and anticipate manufacturing deviations. The proposed integration of QbD, PAT, and AI into a unified digital framework represents a forward-looking model for smart, scalable, and compliant biosimilar production.

Finally, this research expands the discussion towards global access and patient-centered care, highlighting how innovations in excipients, subcutaneous delivery, immunogenicity monitoring, and legal foresight (e.g., freedom-to-operate assessments) contribute to the creation of more accessible and sustainable biopharmaceuticals. Together, this article not only summarizes current advances but also proposes a visionary AI-based roadmap for next-generation biosimilar development: one that is safer, more efficient, and strategically positioned to impact global health.

To consolidate the multidimensional analysis presented throughout this review, Table 8 provides a summative overview of the major formulation challenges associated with biosimilar development, along with the corresponding strategic innovations, enabling tools or technologies, and their regulatory implications. This table illustrates the convergence of pharmaceutical formulation, regulatory frameworks, and digital enablers, reinforcing the core argument of the article that biosimilar development requires a multidisciplinary, system-level approach.

### Future Research Directions

Looking ahead, continued evolution and innovation will define the future of biosimilar development and will likely focus on refining analytical and predictive capabilities. For example, next-generation analytical assays and bioinformatics tools can be used to accurately characterize even more complex biologics (such as bispecific antibodies or gene therapy vectors).

AI is expected to significantly transform biosimilar production through enhanced predictive analytics, real-time process control, and in silico optimization throughout the production pipeline [1,2]. AI systems are capable of processing and integrating multi-omics data, formulation parameters, and manufacturing conditions to streamline critical activities such as cell line development and bioprocess optimization. This innovation aims to streamline processes such as cell line development and bioprocess optimization, potentially reducing the time to market for biosimilars [3,247]. Machine learning (ML) contributes by analyzing complex bioprocess data to identify deviations, enabling proactive corrective actions that align with the QbD framework [349]. This proactive analysis allows the anticipation of potential process deviations before they cause significant problems, facilitating adaptive control measures and ultimately leading to fewer production failures [307]. By continuously analyzing large datasets, ML algorithms can detect subtle variations in critical process parameters such as pH changes, metabolite levels, and protein aggregation tendencies.

In terms of global access to biologic therapies, innovations in biosimilars, including cost-effective production methods and adaptable manufacturing models, can significantly lower costs [1,2]. Technologies that enable high-yield expression systems, low-cost single-use bioreactors, and thermostable formulations contribute to reduced production costs [52]. Moreover, advances in decentralized manufacturing, facilitated by digital twins and AI-driven platforms, allow for the establishment of localized biosimilar production facilities in emerging markets [350]. These advances may facilitate local production capabilities, promoting greater availability in low-resource regions [63,348,351]. Regulatory policies that encourage the development of biosimilars also play a critical role in democratizing access to advanced therapies, resulting in substantial savings and improved patient outcomes in various healthcare settings [352]. This local production capacity not only democratizes access to essential biologic therapies but also addresses healthcare disparities by making effective treatments more affordable [106]. Regulatory bodies are increasingly aligning their frameworks to support these innovations, fostering an environment conducive to the accelerated adoption of biosimilars on a global scale, which is crucial for improving patient access and reducing healthcare costs [259].

AI and machine learning are poised to play a pivotal role, from optimizing cell line development and production processes to personalizing treatment strategies based on big data. Adopting these digital technologies offers the opportunity to shorten development cycles and proactively ensure quality, ushering in an era of ‘digital biotechnology’ where virtual models and real-time analysis guide much of the development process. On the regulatory front, further global harmonization of biosimilar guidelines is anticipated, and potentially the emergence of regulatory frameworks for new classes of biosimilars (e.g., interchangeable biosimilars in more regions or pathways for cell and gene therapies with biosimilars in the future, greater use of recombinant gene therapies). Collaboration between regulatory agencies can generate unified standards that simplify approval and pharmacovigilance globally, further facilitating market access. From a manufacturing perspective, the trend is toward automation, continuous processing, and smart factories, improving specificity, scalability, and cost-effectiveness. These advances could expand the range of biological products that can be developed as biosimilars, including more complex and sensitive molecules. Importantly, addressing the psychosocial aspect will remain a priority. The next phase in biosimilar development offers numerous opportunities through innovation, while maintaining a commitment to scientific rigor and embracing digital technologies, which open doors to great potential. These efforts will strengthen the dual promise of biosimilars: providing equitable access to advanced therapies for patients worldwide while maintaining the highest standards of quality and therapeutic efficacy.

## Figures and Tables

**Table 1:** Differences between chemical, biological, generic, and biosimilar drugs.

Parameter	Chemical Drug	Generic Drug	Biological Drug	Biosimilar Drug
Synthesis	Production of original chemical formula	Copy of the original chemical formula	Insertion of a gene into a cell clone	Development derived from the original biological molecule
Size	100–1000 Da	100–1000 Da	10,000–300,000 Da	10,000–300,000 Da
Glycosylation process	Zero	Zero	Several	Several
Molecular structure	Simple	Simple	Complex	Complex
Ability to generate immunity	Low	Low	Medium-high	Medium-high
Drug development time	7–10 years	1–3 years	10–15 years	6–9 years

Adapted from [1].

**Table 2:** Detailed analysis and proposal of inclusion and exclusion criteria.

**Type of Inclusion Criteria**	**Details**
Relevance of the topic	Studies in biopharmaceutical and biosimilar formulation strategies in the industry, with an emphasis on buffer-free systems.
Formulation technologies, biosimilar design, and trends focusing on FDA and EMA regulations	Specific articles on the formulation of biosimilars to improve their accessibility, efficacy, and safety, highlighting buffer-free systems, safe excipients, intellectual property challenges, and the use of QbD, PAT, and artificial intelligence to optimize the regulated (FDA and EMA) and competitive development of biologic therapies.
Scope of the study	To provide an integrative review of biosimilar formulation strategies, with emphasis on buffer-free systems, safety classification, regulatory alignment, and intellectual property considerations to guide future development and market access.
Type of publication	Articles in peer-reviewed journals, systematic reviews, and reports from reputable institutions and websites.
Period of time	Publications primarily from 2018 to 2025. This range of years was specifically selected to include recent events related to biosimilar formulation. strategies, with an emphasis on buffer-free systems. Older studies will only be included to understand specific topics.
**Exclusion Criteria**	**Details**
Irrelevant topics	Studies did not directly focus on the selected keywords.
Limited crisis context	Articles that do not analyze the formulation of biosimilars, their accessibility, efficacy, and safety, bufferless systems, safe excipients, intellectual property challenges, and the use of QbD, PAT, and artificial intelligence to optimize the regulated (FDA and EMA) and competitive development of biological therapies.
Lack of regional and global focus	Research that focuses solely on localized issues without connecting them to the scope of this research.
Low credibility	Non-peer-reviewed articles, opinion pieces, or studies with unverifiable data.
Duplicate or overlapped content	Studies with existing duplicate results are not considered valid unless they offer new insights or updates.
Language/accessibility	Exclude studies that are not available in English unless accessible translations are provided or accessible for systematic review.

**Table 3:** QbD–PAT–AI Integration: Explanation of the flow elements of Figure 2.

Step	Explanation
Definition of the Quality Target Product Profile (QTPP)	High-level product goals: route, dosage, stability, etc.
CQAs	Measurable attributes that affect safety and efficacy (e.g., aggregation, viscosity)
Apply Risk Assessment	Use FMEA or Pareto to identify high-risk parameters
Design of Experiments (DoE) using QbD tools	Use MODDE^®^, JMP^®^, or Design-Expert^®^ to systematically vary inputs
Real-Time Data Collection Using PAT sensors	Real-time sensing: Raman, NIR, and FTIR for critical variables like pH and osmolality
AI/ML models analyze PAT + historical data	Platforms like BioPharma Finder™, TensorFlow, or DeepChem predict trends
Predictive Modeling of Formulation Outcomes	Forecast viscosity, stability, and bioequivalence from design space
Optimization of formulation parameters	Fine-tune concentrations, pH, excipients
Refinement of the verification and control strategy	Based on process capability and design space robustness
Regulatory submission with integrated QbD-AI evidence	Aligned with ICH Q8–Q11 and regulatory AI guidance (FDA/EMA evolving).

**Table 4:** Biosimilars derived from recombinant therapeutic proteins.

Biosimilar Name	Reference Biologic	Disease/ Condition	FDA Approval Year	Key Findings/Insights	References
Zarxio (filgrastim-sndz)	Neupogen (filgrastim)	Neutropenia	2015	The first biosimilar was approved in the US. Comparable efficacy and safety to Neupogen.	[167]
Ontruzant (trastuzumab-dttb)	Herceptin (trastuzumab)	Breast Cancer	2019	Demonstrated non-inferiority to Herceptin in terms of efficacy.	[51,168]
Amsparity (bevacizumab-maly)	Avastin (bevacizumab)	Various Cancers	2017	Similar safety and efficacy profile as Avastin, improving the accessibility of treatment.	[169]
Amgen’s Amjevita (adalimumab-atto)	Humira (adalimumab)	Rheumatoid Arthritis	2016	The first biosimilar approved, adalimumab, reported comparable safety and efficacy.	[170,171]
Kanjinti (trastuzumab-anns)	Herceptin (trastuzumab)	Breast Cancer	2019	Validated efficacy through phase III trials, expanding treatment options.	[168]
Breztri (bococizumab)	Repatha (evolocumab)	Hyperlipidemia	2021	Efficacy and safety profile similar to those of Repatha, although more data could be beneficial.	[167]
SB5 (adalimumab)	Humira (adalimumab)	Inflammatory Bowel Disease	2021	High similarity in clinical effectiveness observed in real-world IBD cohorts.	[172]
Remsima (infliximab)	Remicade (infliximab)	Rheumatoid Arthritis	2013	Known for cost-effectiveness; Comparable efficacy in patients transitioning from Remicade.	[173,174]
Neulasta Onpro (pegfilgrastim-jmdb)	Neulasta (pegfilgrastim)	Neutropenia	2018	Shows equivalent safety and efficacy to Neulasta, supporting oncology protocols.	[167]
MYL-1501D (insulin glargine)	Lantus (insulin glargine)	Diabetes	2019	Indicated for type 1 diabetes; demonstrated comparable safety profiles.	[175]

**Table 5:** Common excipient types in therapeutic protein formulations and their functions.

Excipient Class	Function in the Formulation	Examples
Buffers	Maintain a stable pH environment	Histidine, phosphate, acetate, citrate, etc.
Sugars/polyols	Stabilize protein conformation (lyoprotectant or bulking agent)	Sucrose, trehalose, mannitol
Amino acids	They are stabilized by charge or hydrophobic interactions; sometimes they act as weak buffers or tonicity agents.	Arginine, glycine, proline, histidine
Surfactants	Prevent surface-induced aggregation (e.g., agitation, interface)	Polysorbate 80, Polysorbate 20
Chelating agents	Preventing metal ion-induced degradation	EDTA, DTPA
Antioxidants	Protect against protein oxidation.	Methionine, Glutathione
Tonicity modifiers	Adjust osmolality to the physiological range	Sodium chloride, glycerol

Adapted from [193].

**Table 6:** Regulatory criteria and evidence requirements for biosimilar formulation differences: a comparison of FDA and EMA frameworks.

Aspect	FDA Approach	EMA Approach	Representative References
General Stance on Formulation Differences	Allows formulation differences, particularly in clinically inactive components (excipients), as long as safety, purity, and potency remain equivalent to the reference product.	Permits formulation differences if justified scientifically and clinically. Acceptable if no detrimental effects are introduced.	[2,66,171,271,272,273,274]
Use of Different Excipients (Clinically Inactive Components)	The differences allowed must be justified through analytical data and often clinical studies. Caution is advised if the excipients interfere with the analytical comparability.	Minor differences are acceptable; must be justified. The avoidance of new excipients is preferred unless safety is demonstrated.	[2,3,271,275,276,277]
Use of New Excipients or Novel Routes	Additional safety/toxicological data are expected for new excipients or excipients used via a new route.	New excipients not previously used in biology are discouraged. If included, prior safety data or similar precedent is required.	[3,244,278]
Justification Requirements	Sponsors must demonstrate that they have no impact on safety, purity, or potency using validated methods.	A complete scientific rationale and risk mitigation must be submitted for formulation differences.	[2,117,171,178]
Encouragement of Formulation Innovation	Encourage the use of advanced, cutting-edge formulation technologies if biosimilarity is preserved. Avoid imposing outdated reference formulations.	Supports formulation updates aligned with patient needs (e.g., improved tolerability, stability), but changes must be scientifically justified.	[2,53,271,272,273,274]
Analytical Comparability of Interference Risk	Formulations should allow robust analytical comparability. Excipients that hinder testing are discouraged. Requires assurance that excipients do not interfere with analytical comparability assays.	Demands evidence that changes in excipients do not affect comparability studies.	[209,275,276]
Changes in Dosage Form (e.g., liquid vs. lyophilized)	Permitted if the safety, purity, and potency remain consistent and the differences are justified.	Allowed if justified. The route of administration must remain the same; any change in dosage form must not affect product performance.	[273,274]
Regulatory Expectation for Documentation	Differences must be clearly documented and justified in the application.	All formulation differences are reviewed and discussed in EMA assessment reports, with a clear justification.	[272,275]
Clinical Testing Requirement	It is necessary to see if formulation changes could affect PK/PD or clinical response.	Clinical studies are required when formulation changes could affect bioavailability or tolerability.	[178,257]
New Dosage Forms	Permits with evidence of unchanged safety and efficacy.	Permits if the route is unchanged and product performance is validated.	[53,178]
Documentation Practices	Comprehensive submission required with full rationale and evidence for any formulation changes.	Extensive assessment reports document and justify all formulation differences.	[171,275]
Examples of Acceptable Changes	Omission of antimicrobial preservatives in single-dose vials; inclusion of newer and safer excipients with full justification.	Similar cases accepted; removal of preservatives in single-use formats or omission of unnecessary excipients. Emphasis on safety, patient convenience, and technological justification.	[271,275]

**Table 7:** IP Challenges and Strategic Formulation Recommendations for Biosimilars.

Aspect	Challenge/Strategy	Description and Examples
Patent Thickets	Freedom-to-operate (FTO) analysis	Biosimilar developers map reference product patents and analyze expiry timelines and claim breadth. May delay launch if critical patents expire soon. Use of inter-patent review IPR (US) or opposition (EU) to challenge weak patents.
Designing Around Patents	Formulation modifications	Launch with legacy formulations to avoid infringement (e.g., citrate buffered adalimumab). Switch to citrate-free after the expiration of the patent. Biosimilars of insulin glargine are used in vials instead of pen devices due to device patents.
Patent Multiplicity	Continuations and layered patents	Developers exploit continuation strategies (e.g., in the US) to file overlapping claims. Litigation during Phase 3 can help resolve disputes before launch.
Trade Secrets	Reverse engineering	Some reference formulations remain partly undisclosed (e.g., additives, processes). Biosimilar makers reverse-engineer composition and verify using analytical techniques, but some process steps (e.g., lyophilization) may remain protected.
Formulation Optimization	Use cutting-edge science	Prefer a ready-to-use liquid over lyophilized if stable. Use stabilizers such as trehalose or histidine. Support high-concentration subcutaneous injections if safe. The improvements must be justified with comparability data.
Excipient Risk Minimization	Select safe and known ingredients	Avoid new excipients unless essential. Use animal-free, pharmacopoeial-grade excipients. Minimize concentration and number of excipients to reduce the risks of immunogenicity and toxicity.
Pharmaceutical Equivalence	Match reference where possible	Maintain the dosage form, route, and protein concentration. Modify excipients (e.g., citrate replaced with acetate) to reduce pain but preserve administration parameters. Justify changes with data.
Stability and Compatibility	Comprehensive testing required	Evaluate under stress conditions (e.g., freeze-thaw, light, agitation). Ensure compatibility with containers and infusion hardware. Prevent protein aggregation or crystallization.
Patient-centered Design	Optimize for Comfort and Usability	Reduce injection pain (e.g., avoid citrate/glutamate), improve convenience (e.g., tolerate room temperature). Consider auto-injectors, thinner needles, or reduced injection frequency.
IP-Informed Development	Align Formulation with IP Landscape	Identify and avoid violating excipient combinations. If infringement is unavoidable but preferable, plan for post-expiration launch or license negotiation. Legal safety should not compromise patient safety.
Regulatory Engagement	Early Agency Dialog	Proactively discuss formulation deviations with FDA/EMA. Use scientific advice or type IV meetings. Emphasize patient safety and similarity to gain support for changes.

**Table 8:** Summative Overview of Key Challenges and Strategic Innovations in Biosimilar Formulation.

Formulation Challenge	Strategic Innovation	Tools/Technologies	Regulatory Relevance
Protein Aggregation and Viscosity	Buffer-free high-concentration systems	QbD, AI simulation, Ensilication	Reduces immunogenicity risk
Post-Translational Modifications (PTMs)	AI-assisted PTM mapping and Deep Learning prediction	GlycoAnalytics™, Mass Spectrometry, AI	Ensures biosimilarity of functional domains
Regulatory Equivalence	Totality of evidence approach	QbD–PAT–AI integrated systems	EMA/FDA stepwise biosimilarity validation
Legal Barriers (IP Thickets)	Early Freedom-to-Operate (FTO) and Formulation Design	Patent Landscape, AI-FTO Analysis Tools	Minimizes litigation, secures market access
Patient Tolerability	Citrate-free excipient-optimized subcutaneous delivery	AI-enhanced excipient selection	Enhances compliance and real-world outcomes

## Data Availability

No new data were created or analyzed in this study. Data sharing is not applicable to this article.

## Appendix A

Table A1, through a summary of the latest advances and associated challenges faced by biosimilar developers, allows us to observe some aspects directly related to the design, manufacturing, and analytical characterization of biosimilars. Each row of Table A1 focuses on a specific domain, describing its relevance and the most advanced improvements found, highlighting innovative strategies from molecular design to AI integration. These interconnected advances represent a roadmap that guides future research, regulatory approaches, and industry practices in biopharmaceutical innovation.

**Table A1:** Aspects directly related to the design, manufacturing, and analytical characterization of biosimilars.

Aspect	Description	The Last Insinuations	Challenges	Refserences
Design and formulation of strategies	Molecular design and formulation optimization replicate the stability, efficacy, and immunogenicity of reference biologics through molecular engineering, innovative excipients, and stability enhancements.	Innovative formulation strategies including high-concentration and buffer-free systems. AI-driven computational design and modeling (critical attribute matching) for excipient selection and aggregation minimization Alternative excipients (arginine) reduce immunogenicity. Nanomedicine approaches for greater stability.	Complex reproduction of PTM. Maintain structural consistency and bioavailability. Maintain microheterogeneity and stability. Replication of complex PTMs. Immunogeneticity induced by variability.	[2,148,209,353,354,355]
Cell line and upstream engineering	Development and optimization of cell lines to ensure high similarity with the attributes of the reference product. Production of recombinant proteins using systems such as CHO cells, crucial for protein structure and PTMs.	Genomic engineering (CRISPR) to optimize performance and glycosylation. Continuous improvements to the cell culture process and media optimization. Optimized cell lines and continuous processing. Digital monitoring and dielectric spectroscopy for performance optimization.	Variability of expression and impurities. PTM profiles of engineered cell lines. Consistency challenges in scaling.	[356,357,358,359,360,361]
Improvement processes and PAT	Protein purification and recovery that ensures removal of impurities, integrity of protein folding/refolding, and consistency of the product. Real-time monitoring/control through PAT, ensuring QbD.	Advanced chromatography (multimodal, UF/DF ultrafiltration), single-use modular systems. Continuous processing with advanced PAT for impurity monitoring. Refolding techniques that preserve protein integrity. Predictive machine learning analytics for real-time adjustments.	Variability during scaling in impurity profiles. Removal of impurities from the host cell. Translation of purification processes from R&D to commercial scale. Aggregation control. Reproducibility of batches and scale-up. Integration of PAT into legacy systems. Robustness of data for regulatory compliance.	[53,57,361,362,363]
Analytical Characterization and Bioassays	Structural and functional evaluation using orthogonal methods to establish biosimilarity throughout the body of evidence.	Surface plasmon resonance (SPR) for protein-receptor interactions. Advanced LC-MS and robust cell-based bioassays for bioactivity and potency assessments. MRI 2D, glycoprofiling and bioassays. Omics-Driven Biomarker Analysis and In silico Predictive Models.	Detect subtle structural differences that impact clinical outcomes. Link analytical differences with clinical efficacy.	[37,188,364,365,366,367,368,369]
Regulations and Harmonization	Regulatory evaluations that ensure the safety, efficacy, and quality of biosimilars through comparability studies, ensuring that biosimilars comply with the strict FDA/EMA regulatory frameworks.	‘Total evidence’ approach of the FDA and EMA, with an emphasis on analytical data. Harmonized comparability studies and accelerated pathways that leverage analytical data. Incorporation of real-world evidence for risk-based approvals.	Regional regulatory variations and broad demands for comparability data. Generation of comprehensive data without excessive clinical trials. Balance between costs and time between clinical and analytical tests.	[5,37,168,273,370,371]
IP, Innovation Strategy, and Market Access	Patents related to formulation, manufacturing, and analytical techniques that affect market entry and competitiveness.	Innovations in patented (expired) formulations and manufacturing processes. Analyze freedom of operation and strategic legal partnerships.	Patent ‘tangles’ that cause delays in market entry. Continuous innovation while maintaining biosimilarity. Litigation creates the risk of increasing development time and costs.	[371,372,373]
Integration of Digital Tools and AI	Application of digital platforms, AI, and simulations to optimize biosimilar development.	Predictive modeling with AI for process optimization (e.g., glycosylation control). Simulators of in silico clinical trials that reduce dependence on extensive in vivo studies.	Complexity of high-dimensional data integration. Transparency and reproducibility of AI models for regulatory compliance.	[374,375]
